# Research funding challenges in Brazil: researchers' perceptions from a public institution of professional education

**DOI:** 10.3389/frma.2025.1553928

**Published:** 2025-09-22

**Authors:** Cristhian Chagas Ribeiro, Woska Pires da Costa, Marcos de Moraes Sousa, Priscilla Rayanne E. Silva, Vicente Miñana-Signes, Matias Noll

**Affiliations:** ^1^Instituto Federal Goiano - Campus Ceres, Ceres, Brazil; ^2^Universidade Estadual de Goiás (UEG) - Campus Porangatu, Porangatu, Brazil; ^3^Instituto Federal Goiano - Campus Morrinhos, Morrinhos, Brazil; ^4^Universitat de València, València, Spain; ^5^Universidade Federal de Goiás (UFG), Goiânia, Brazil

**Keywords:** research funding, public funding, funding agencies, gender-equal funding, vocational and technological education, scientific ecosystem, scientific policy, research trends

## Abstract

**Introduction:**

In a global landscape characterized by intense competition and stringent funding criteria, researchers face the dual challenges of limited resources and high demand for innovation—a challenge that Brazil is no exception to. This study aimed to explore the perceptions, barriers, and challenges faced by researchers during the project submission process for approval by funding agencies, with a focus on schools within the Federal Network of Professional, Scientific, and Technological Education Institutions.

**Methods:**

A quantitative cross-sectional approach was used to examine the characteristics of researchers at a Brazilian institution in 2023. The sample comprised eighty three researchers who completed an online questionnaire containing eighty three questions on demographic characteristics, factors associated with project submission and approval, and reasons for non-submission or non-approval. The data were analyzed using descriptive statistics, including the Kolmogorov—Smirnov, Pearson's chi-square, and Mann—Whitney *U*-tests, followed by *post hoc* analysis and Yates' correction. Logistic regression was applied using the backward elimination method, and significant parameters (*p* < 0.20) free from multicollinearity were selected.

**Results:**

This study revealed that most researchers were men (61.4%) with doctoral degrees (91.6%), highlighted the critical role of proposal clarity and relevance in the project evaluation process. Gender (*p* = 0.011) and academic level (*p* = 0.025) were significant factors influencing project submission rates, with Brazilian National Council for Scientific and Technological Development (*CNPq*) fellows and researchers involved in graduate programs submitting more projects. The participants identified “search for funding” and “desire to expand research impact” as their primary motivations while citing “complex funding calls” and “funding limitations” as major barriers. Additionally, age and the number of children were found to affect project approval (*p* ≤ 0.018), with “proposal clarity” and “researchers' experience” having been critical factors for submission approval (*p* ≤ 0.03).

**Conclusion:**

The study results highlighted a gender disparity, with lower participation among women, and identified key factors influencing project submission, including the search for funding, curriculum development, and structural challenges. Additionally, the findings suggest the adoption of gender-sensitive and early-career grant criteria, targeted support for underrepresented researchers, and flexible mechanisms for those with caregiving responsibilities. These findings underscore the importance of public policies and institutional strategies in promoting equitable and inclusive funding opportunities.

## 1 Introduction

Research is a dynamic and challenging field in which scholars, scientists, and organizations vie for a limited funding pool to support their studies (Fanelli, 2010). This competition is driven by the pursuit of new knowledge and technological innovation and the need to address local and global challenges (Lane and Bertuzzi, 2011; Maad, 2017). Funding for scientific and technological research is crucial, as it enables the acquisition of equipment, the retention of qualified personnel, the execution of experiments, and the dissemination of results (Baczkiewicz et al., 2020). Although various funding sources are available—including government grants, private-sector investments, and philanthropic contributions—increasing hyper competition within the scientific community has intensified the selectivity of funding processes (Tsugawa et al., 2022; Garcia and, 2005). This competitive environment stimulates researchers to innovate and tends to favor well-established researchers and institutions with strong track records, often making it particularly challenging for early-career researchers and newcomers to secure funding (Lane and Bertuzzi, 2011).

In many cases, the difficulty lies not in identifying where funding opportunities are but in overcoming the structural barriers imposed by competition with more prominent researchers (Wang et al., 2018), who have greater visibility, established networks, and proven outcomes. This situation underscores the importance of developing grant-writing skills and applying for career-stage-appropriate funding, such as training grants or early-career fellowships, to increase competitiveness. Nevertheless, challenges persist, including the pressure to align research with areas perceived to have higher economic returns rather than basic science (Sridhar, 2012; Daumann et al., 2023). Consequently, the dynamics of funding distribution have long-term implications for scientific progress, knowledge production, and society's ability to address its most pressing challenges.

Submitting and approving original research proposals are essential steps for researchers seeking financial support. Multiple factors influence these processes, shaping the researcher's decision to apply for funding and the probability of success in competitive public calls. Several studies have explored these dynamics in depth (McManus et al., 2021; Kataeva and DeYoung, 2018; Furukawa and Olm Cunha, 2010; Francisco and Zucatto, 2019; Bol et al., 2022; Neema and Chandrashekar, 2021; Souza et al., 2020). Funding decisions are typically based on a combination of strict peer review criteria, including the researchers‘ characteristics, the quality and innovative capacity of the proposal, its potential impact, feasibility, researchers' skills (Hug and Aeschbach, 2020), and methodological soundness.

Structural factors—such as research knowledge area, career stage, and gender—influence the likelihood of submitting proposals and achieving funding success. For example, evidence suggests that early-career researchers and women may submit fewer proposals and experience lower success rates due to structural barriers (Rusu et al., 2022) and accumulated disadvantages in the research ecosystem. Moreover, securing funding is often a prerequisite for conducting research, leading to robust scientific outputs, including publications and citations (Heyard and Hottenrott, 2021). While access to funding can facilitate greater productivity, it does not inherently guarantee research quality, which is influenced by various factors beyond financial resources (Heyard and Hottenrott, 2021). Nevertheless, a cumulative advantage persists, whereby researchers who have already obtained funding are more likely to secure new resources, reinforcing disparities (Bol et al., 2022) and making it difficult for early-career researchers to overcome this barrier.

In addition, other factors such as the implementation plan, the social and economic relevance of the research topic, the technical and academic skills, the infrastructure available, compliance with ethical and legal requirements, and alignment with the funding agency's priorities, are considered in the research project evaluation process (Kataeva and DeYoung, 2018). In the Brazilian context, for example, researchers face additional challenges closely tied to the funding landscape, including excessive bureaucracy in grant management, insufficient administrative and institutional support, limited infrastructure, and an underdeveloped culture of collaborative networking among researchers and institutions (McManus and Baeta Neves, 2021; McManus et al., 2021)—all of which can constrain both the submission of competitive proposals and the successful execution of funded research.

Considering this landscape, this study focused on the experiences of researchers from a federal institute of education, science, and technology—a typical Brazilian public institution that combines academic, vocational, and technological education—in the State of Goiás, Brazil. The context is relevant since some funding lines from agencies are directed explicitly toward certain institutions, regions, or strategic areas, which directly affects access to research funding in Brazil. Thus, this study aimed to investigate researchers' perceptions of the barriers and challenges faced during the research project submission process to funding agencies, including project submission and approval. This study contributes to the international debate on the elements influencing funding by offering insights for developing more effective public policies and strategies that consider the specific characteristics of institutions and the research ecosystem. Furthermore, understanding how submission and approval outcomes are influenced, among other factors, by demographic characteristics and the reasons for submission, non-submission, approval, or rejection of research proposals provides valuable evidence to support informed decision-making by funding agencies and institutional managers.

## 2 Methods

A quantitative cross-sectional study, part of the umbrella study “Submission and Approval of Research Projects to Funding Agencies in Brazil” (SARFA-Bra study), was conducted.

### 2.1 Context

The study was conducted at the *Instituto Federal de Educação, Ciência e Tecnologia Goiano* (*IF Goiano*), a public institution with twelve campuses and an innovation hub spread across several cities in the state, with its administrative headquarters located in Goiânia, the capital of Goiás. The *IF Goiano* is part of the Federal Network of Professional, Scientific, and Technological Education Institutions (*RFEPCT*), which is represented in all Brazilian states and is the cornerstone of the country's scientific and technological progress (Brazil, 2008; IF Goiano, 2023; Melo et al., 2023). This institution offers from high school education to doctoral programs (Melo et al., 2023; Galvão et al., 2022; Costa et al., 2024).

### 2.2 Population and sampling

The target population consisted of *IF Goiano* researchers involved in undergraduate scientific, technological, and innovative research projects, or, in a strict sense, graduate programs, as of 2022. Among the 327 participants, 83 completed the questionnaire, resulting in a response rate of 25.4%.

### 2.3 Research ethics

This study was approved by the Human Research Ethics Committee (*CAAE* No. 67695523.4.0000.0036, Opinion No. 6.144.987 on June 27, 2023). The participants were guaranteed anonymity, the right to withdraw from the study at any time, and the freedom not to complete the questionnaire. An Informed Consent Form (ICF) was signed electronically before administering the questionnaire.

### 2.4 Data collection instrument

The data were collected through the online “*IF Goiano* Researcher's Profile” questionnaire (Supplementary Materials 1, 2—original and English-translated versions, respectively). This instrument consisted of one open-ended question and one hundred and forty seven closed-ended questions, logically organized to record participants' respective experiences. The analyzed parameters were divided into the following main blocks: (1) sociodemographic data, (2) work-related data, (3) factors associated with project submission, (4) factors associated with project approval, (5) factors associated with project non-submission, (6) factors associated with project non-approval, (7) factors associated with project submission and approval, (8) Brazilian National Council for Scientific and Technological Development (*Conselho Nacional de Desenvolvimento Cient*í*fico e Tecnológico – CNPq*) productivity fellowship, and (9) family structure and the impact of parenthood.

### 2.5 Data collection procedures

Data were collected through a self-administered online questionnaire sent electronically to the target population. The participants were invited via email, and their confidentiality and anonymity were ensured. Data collection took place between August 17 and October 6, 2023, following approval of the study by the Research Ethics Committee, thereby ensuring compliance with the ethical guidelines for human research.

### 2.6 Data analysis

The sample was characterized via descriptive statistics. The Kolmogorov—Smirnov test was used to assess the normality of the data. Pearson's parametric chi-square test (χ^2^) was used to associate the sample profile with project submission and approval (Koehler, 2005). Contingency tables greater than 2 × 2, presenting statistically significant differences, were subjected to *post hoc* analysis of standardized residuals to determine which contingency cell was significantly different, as proposed by MacDonald and (2000). Yates's correction was applied to chi-square cells with an expected absolute frequency of < 5.

Continuous scales were compared with project submission and approval using the Mann—Whitney *U*-test. Statistically significant parameters were included in the hierarchical multivariable logistic regression analysis by selecting parameters with a *p*-value of less than 0.20 in univariate exploratory analyses, ensuring no multicollinearity (variance inflation factor—VIF test). A threshold of *p* < 0.20 was adopted for variable selection in the multivariate model, based on established methodological recommendations that advocate for a more inclusive threshold during the exploratory phase to reduce the risk of prematurely excluding potentially relevant predictors. This approach enhances the model's sensitivity and ensures that important variables are retained for more rigorous analysis in the multivariate phase (Bursac et al., 2008; Bendel and Afifi, 1977). The collected dataset was analyzed using the Statistical Package for the Social Sciences (IBM™ SPSS™ Statistics) software, version 26.0 for Microsoft Windows™ (IBM Corp., Armonk, NY, USA).

## 3 Results

These findings were based on responses from researchers (*N* = 83) who completed an online questionnaire. Most researchers were male (61.4%), aged 34–39 years (28.9%), self-identified as white (53.0%), in a relationship (73.5%), and had two children (46.2%). Most had a doctoral degree (91.6%), worked in graduate programs (49.4%), had up to 10 years of research experience (63.9%), and participated in research projects (96.4%). However, only 10.8% had received a *CNPq* productivity fellowship, a grant awarded to recognize and support outstanding researchers for the excellence and impact of their scientific output (Table 1).

**Table 1:** Distribution of research projects submitted according to the researchers' profiles.

**Parameters**	***n* (*%*)**	**Submitted projects**				p^*^
		**None**	**1–3**	**4–6**	≥**7**	
**Age group**						
28–33 years	16(19.3)	6(37.4)	8(50.0)	1(6.3)	1(6.3)	0.515
34–39 years	24(28.9)	6(25.0)	8(33.4)	5(20.8)	5(20.8)	
40–45 years	22(26.5)	5(22.7)	7(31.8)	8(36.4)	2(9.1)	
≥46 years	21(25.3)	4(19.0)	8(38.1)	5(23.8)	4(19.0)	
**Gender identification**						
Female	32(38.6)	11(34.4)	16(50.0)	2(6.2)	3(9.4)	**0.011**
Male	51(61.4)	10(19.6)	15(29.4)	17(33.3)≠	9(17.7)	
**Ethnicity**						
White	44(53.0)	10(22.7)	15(34.1)	10(22.7)	9(20.5)	0.417
Pardo/Black	39(47.0)	11(28.2)	16(41.0)	9(23.1)	3(7.7)	
**Marital status**						
With partner	61(73.5)	14(23.0)	24(39.3)	14(23.0)	9(14.8)	0.859
Without partner	22(26.5)	7(31.8)	7(31.8)	5(22.7)	3(13.6)	
**Children**						
No	31(37.3)	9(29.0)	13(41.9)	6(19.4)	3(9.7)	0.659
Yes	52(62.7)	12(23.1)	18(34.6)	13(25.0)	9(17.3)	
**Number of children** ^**^						
1	21(40.4)	5(23.8)	7(33.3)	4(19.0)	5(23.8)	0.481
2	24(46.2)	6(25.0)	9(37.5)	5(20.8)	4(16.7)	
3	7(13.5)	1(14.3)	2(28.6)	4(57.1)	–	
**Age of youngest child** ^**^						
≤ 1 year	12(14.5)	5(41.7)	4(33.3)	1(8.3)	2(16.7)	0.367
2–5 years	13(15.7)	4(30.8)	3(23.1)	3(23.1)	3(23.1)	
6–10 years	8(9.6)	1(12.5)	2(25.0)	4(50.0)	1(12.5)	
11–17 years	10(12.0)	–	5(50.0)	2(20.0)	3(30.0)	
>18 years	9(10.8)	2(22.2)	4(44.4)	3(33.3)	–	
**Education level**						
Master's degree	7(8.4)	3(42.9)	2(28.6)	1(14.3)	1(14.3)	**0.025**
Doctorate degree	55(66.3)	17(30.9)	23(41.8)≠	11(20.0)	4(7.3)	
Postdoctoral qualifications	21(25.3)	1(4.8)	6(28.6)	7(33.3)	7(33.3)≠	
**Working in a graduate program**						
No	42(50.6)	18(42.9)≠	20(47.6)	1(2.4)	3(7.1)	**< 0.001**
Yes	41(49.4)	3(7.3)	11(26.8)	18(43.9)≠	9(22.0)	
**Research experience**						
≤ 10 years	53(63.9)	15(28.3)	21(39.6)	11(20.8)	6(11.3)	0.569
>10 years	30(36.1)	6(20.0)	10(33.3)	8(26.7)	6(20.0)	
**Participation in a research project**						
No	3(3.6)	2(66.7)	1(33.3)	–	–	0.350
Yes	80(96.4)	19(23.8)	30(37.5)	19(23.8)	12(15.0)	
***CNPq*** **fellowship holder**						
No	74(89.2)	21(28.4)	30(40.5)≠	16(21.6)	7(9.5)	**0.001**
Yes	9(10.8)	–	1(11.1)	3(33.3)	5(55.6)≠	

The “*n*” values represent absolute frequencies, whereas the “%” values represent relative frequencies. The final sample size was *N* = 83. ^*^p-value for Pearson's parametric chi-square test (χ^2^), and bold indicates a result with a statistically significant difference (α = 0.05); ≠ indicates the *post hoc* test. ^**^This question was answered only by participants who indicated that they had children. *CNPq* is an acronym for the Brazilian National Council for Scientific and Technological Development.

The researchers under study agreed with the project evaluation criteria proposed by funding agencies (Supplementary Material 6). A high proportion of respondents expressed agreement with the project evaluation criteria proposed by funding agencies, selecting either “strongly agree” or “agree” on the Likert scale. Specifically, proposal clarity (83.2%), relevance and originality (85.6%), researcher experience (90.4%), collaboration (89.2%), and compatibility with agency priorities (90.4%) were perceived as important and appropriate criteria for evaluation. Methodological design (78.3%), resource availability (73.5%), and ethical compliance (74.7%) had lower agreement rates, with mean responses ranging from 1.66 to 2.12. These results indicate that, despite broad acceptance of most criteria, researchers express some concern about resources and support for methodological and ethical demands.

Among the 83 respondents, 62 had submitted research projects in response to funding calls in the previous 5 years. Of these, 20 had no approved projects, and 42 had at least one approved project. This represents an approximate submission rate of 74.7% and an approval rate of 67.7% among the researchers who had submitted projects (Supplementary Material 3, 4).

Men submitted more projects than women did (*p* = 0.011). The subgroup of doctoral and postdoctoral researchers submitted more projects than the subgroup of other researchers, especially in the categories of four to six or seven or more projects, respectively (*p* = 0.025) (Table 1). These results suggest that advanced academic qualifications and career progression both influence researchers' motivation to submit proposals to funding calls. Furthermore, involvement in graduate programs and being a *CNPq* fellow were associated with higher submission rates (*p* < 0.001 and *p* = 0.001, respectively), reinforcing the idea that institutional status and prior recognition influence research activity. Variables such as age, ethnicity/skin color, marital status, number of children, age of the youngest child, and research experience had no significant impact on project submission rates.

In terms of motivation, the primary drivers for project submission were the pursuit of research funding (91.9%), securing more resources for research (62.9%), and expanding research impact (46.8%). These motivations were consistent across different groups, with no significant differences observed (Supplementary Material 4). The main challenges included complex funding calls (54.8%) and insufficient funding opportunities (33.9%). However, difficulties in establishing institutional partnerships were particularly significant among those who submitted 4–6 projects (57.9%), indicating a significant difference (*p* = 0.008). This suggests that while increased submission efforts may expand opportunities, they also heighten challenges in collaboration building.

Project approval rates were also influenced by the number of submissions: researchers who submitted 1–3 projects had a significantly lower approval rate than those who submitted 4–9 projects (*p* < 0.001). This result suggests that higher submission activity may increase the chances of success.

Evaluation of funding agencies criteria, on a scale of 1 to 5—where 1 means “strongly agree” and 5 means “strongly disagree” (Table 2)—revealed that researchers who submitted proposals rated “proposal clarity” and “researchers expertise” as more critical (means of 1.71 and 1.53, respectively) than non-submitters (mean of 2.10, *p* = 0.022; and mean of 2.05, *p* = 0.002, respectively). Additionally, Approved project holders rated “funding availability” and “partnerships” more favorably than those with rejected proposals (means of 1.90 and 1.52, respectively) compared to those with rejected proposals (means of 2.45, *p* = 0.026; and means of 1.95, *p* = 0.019, respectively). These results underscore that access to sufficient funding and collaborative networks play a critical role in the success of applications.

**Table 2:** Evaluation criteria for submitting and approving projects to funding agencies.

**Agreement**	**Submitting projects**			**Approving projects**		
	**No (*****M*** ± ***SD*****)**	**Yes (*****M*** ±***SD*****)**	**p** ^*^	**No (*****M*** ± ***SD*****)**	**Yes (*****M*** ±***SD*****)**	**p** ^*^
Proposal clarity	2.10 ± 0.70	1.71 ± 0.80	**0.022**	1.85 ± 0.93	1.64 ± 0.73	0.428
Project relevance and originality	1.95 ± 0.86	1.68 ± 0.78	0.165	1.90 ± 0.97	1.57 ± 0.67	0.190
Researcher expertise	2.05 ± 0.67	1.53 ± 0.59	**0.002**	1.70 ± 0.73	1.45 ± 0.50	0.236
Project methodological design	2.05 ± 0.59	2.06 ± 0.85	0.814	2.05 ± 0.94	2.07 ± 0.81	0.726
Resources availability	2.14 ± 0.91	2.08 ± 0.86	0.808	2.45 ± 0.94	1.90 ± 0.76	**0.026**
Adherence to ethical guidelines	2.19 ± 0.75	2.03 ± 0.87	0.342	2.25 ± 1.02	1.93 ± 0.78	0.273
Partnerships and collaborations between institutions	1.76 ± 0.70	1.66 ± 0.65	0.571	1.95 ± 0.69	1.52 ± 0.59	**0.019**
Project funding proportion	2.05 ± 0.67	1.95 ± 0.76	0.460	1.90 ± 0.72	1.98 ± 0.78	0.827
Research impact demonstration	1.90 ± 0.77	1.69 ± 0.74	0.211	2.00 ± 0.97	1.55 ± 0.55	0.061
Project/agency priority compatibility	1.95 ± 0.74	1.61 ± 0.58	0.058	1.75 ± 0.72	1.55 ± 0.50	0.335
Project includes S&T popularization	2.29 ± 0.64	2.06 ± 0.83	0.159	2.25 ± 1.07	1.98 ± 0.68	0.428

“*M*” and “*SD*” represent the mean and standard deviation, respectively. The final sample size was *N* = 83. S&T is the abbreviation for science and technology. ^*^p-value for the Mann–Whitney *U*-test, and bold indicates a result with a statistically significant difference (α = 0.05). The mean values range from 1 to 5, with 1 indicating “Strongly agree” and 5 indicating “Strongly disagree”. The lower the mean, the greater the degree of agreement with the statement.

Logistic regression indicated that female researchers were less likely to submit projects, with an Odds Ratio (*OR*) of 0.47 (95% CI: 0.17–0.72; *p* = 0.01), suggesting a potential disadvantage (Table 3). Parenthood also appeared to impact productivity, with researchers who had children showing lower submission, with an *OR* of 0.70 (*p* = 0.05). Proposals with less clarity have an *OR* of 0.68 (*p* = 0.03), and researchers with less expertise have an *OR* of 0.31 (*p* = 0.01), which were also significant predictors of approval likelihood, indicating that less clear proposals and lower levels of expertise were associated with reduced approval chances.

**Table 3:** Project submission with the study's other exploratory parameters.

**Exploratory parameters**	***r^2^***	***B***	**Standard error**	**Wald**	*p* ^*^	**Odds**	**95% CI**	
							**Lower**	**Upper**
**Profile**								
Gender identification (Female)	0.18	−0.76	0.51	2.22	0.01	0.47	0.17	0.72
**Impact of having children on career**								
Scientific production	0.09	−0.36	0.47	0.60	0.05	0.70	0.28	0.84
**Agreement**								
Less clarity of the proposal	0.24	−0.39	0.35	1.21	0.03	0.68	0.34	0.50
Less expertise of the researcher		−1.17	0.45	6.79	0.01	0.31	0.13	0.75

The *r*^2^ values represent the coefficient of determination; the “*B*” column represents the beta coefficient. ^*^p-value for multivariate logistic regression analysis. The CI is abbreviated as the confidence interval. The mean sample size was *N* = 62.

Approval rates varied by the researcher's age group and number of children. Researchers aged 34–39 years had a 94.4% approval rate (*p* = 0.018), and those with one child had an 87.5% approval rate (*p* = 0.007) (Supplementary Material 4). Motivational factors such as seeking funding (*p* = 0.017), strengthening the academic curriculum (*p* = 0.011), and institutional incentives (*p* = 0.045) also influenced the likelihood of project funding approval. Gender, ethnicity, marital status, age of the youngest child, and the number of projects submitted had no significant impact on approval rates.

Most researchers with approved projects submitted two or three proposals before securing funding. Project funding was the most valued type of institutional support. Budget restrictions and complex funding call criteria were associated with project approval (*p* < 0.05) (Supplementary Material 5.)

Multivariate logistic regression analysis of the project approval data revealed significant associations with several factors. Three reasons for submitting projects positively impacted approval: seeking funding (*OR* = 1.15, *p* = 0.01), strengthening the academic curriculum (*OR* = 1.41, *p* = 0.02), and institutional stimulus (*OR* = 1.38, *p* = 0.02). However, guidance (*OR* = 0.44, *p* = 0.03), network collaboration (*OR* = 0.81, *p* = 0.03), and research coordination (*OR* = 0.64, *p* = 0.05) had negative impacts (Table 4).

**Table 4:** Project approval with the study's other exploratory parameters.

**Exploratory parameters**	***r^2^***	***B***	**Standard error**	**Wald**	p^*^	**Odds**	**95% CI**	
							**Lower**	**Upper**
**Reasons for submitting projects to funding calls**								
Possibility of obtaining funding to conduct the project (Yes)	0.17	1.91	1.21	2.46	0.01	1.15	1.01	1.61
Strengthening the academic curriculum (Yes)		0.89	0.68	1.72	0.02	1.41	1.11	1.55
Institutional incentive to submit projects (Yes)		0.98	0.80	1.51	0.02	1.38	1.08	1.79
**Impact of having children on academic career**								
Activities guidance	0.12	−0.83	0.85	0.95	0.03	0.44	0.08	0.52
Networking collaboration		−0.54	0.62	0.92	0.03	0.81	0.54	0.91
Coordination of research projects		−0.44	0.69	0.40	0.05	0.64	0.17	0.71
**Researcher's agreement with agency evaluation criteria**								
Less availability of resources	0.22	−0.68	0.35	3.65	0.06	0.51	0.25	1.02
Fewer partnerships and collaborations		−0.93	0.48	3.83	0.05	0.39	0.15	1.00

The *r*^2^ values represent the coefficient of determination; the “*B*” column represents the beta coefficient. ^*^p-value for multivariate logistic regression analysis. The CI is abbreviated as the confidence interval. The mean sample size was *N* = 42.

**Figure 1:**
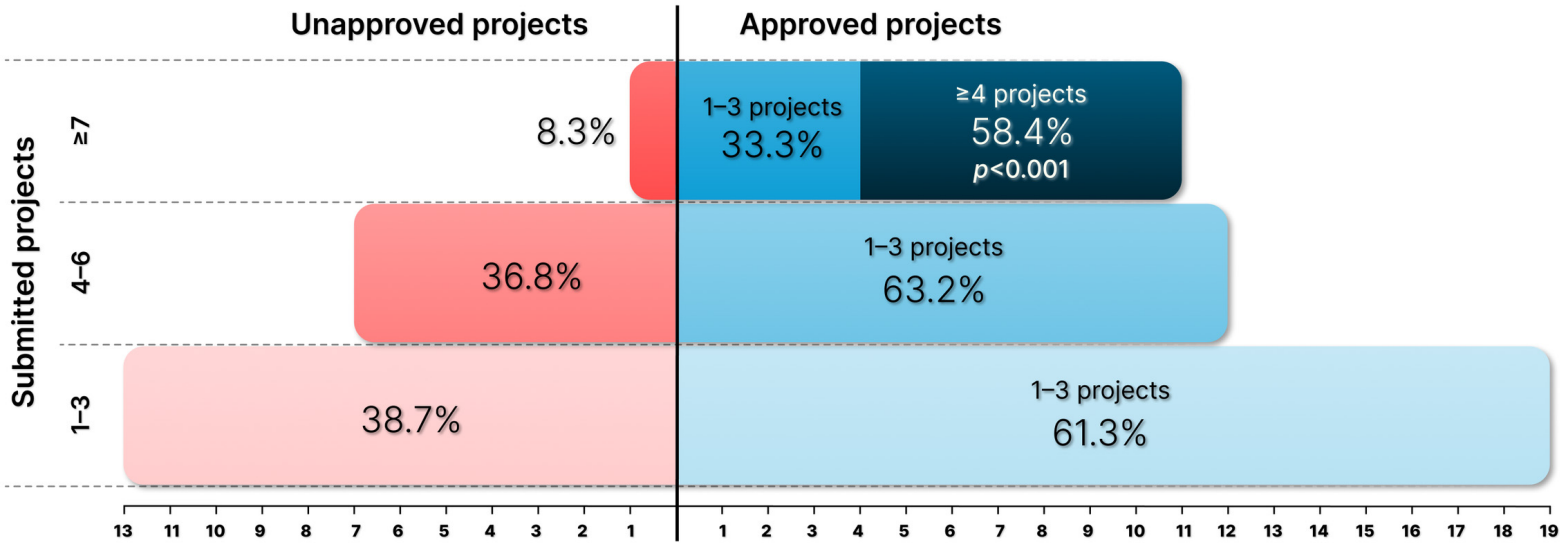
Distribution of approved research projects in terms of the number of projects submitted.

Lastly, the distribution of approved research projects, in terms of the number of projects submitted, is shown by all researchers who submitted projects in Figure 1. This visualization shows that the likelihood of approval increases with the number of submissions.

## 4 Discussion

This study revealed the complex interactions between sociodemographic features and the perceptions, difficulties, and challenges encountered by researchers at a Brazilian Federal Institution—including federal research and professional education institutions and public universities in the Brazilian context—during the project submission process for approval by funding agencies. Our results provided evidence of gender disparities. Factors such as seeking funding and strengthening the academic curriculum were key motivators, whereas structural challenges, including complex funding calls for proposals—i.e., those with extensive bureaucratic requirements—and short deadlines, represented significant barriers.

By integrating these findings with the existing literature, we emphasize the need for more inclusive public and institutional policies—meaning both broader governmental policies that foster research development and internal policies within institutions to facilitate researchers' engagement with funding processes. Additionally, the implementation of effective support mechanisms—such as administrative assistance for proposal submission, guidance on funding calls, capacity-building workshops, and access to research infrastructure. These concepts are essential to help researchers overcome the identified challenges. Together, these actions contribute to enhancing the quality of submissions, increasing approval rates, and promoting more equitable and sustainable research practices.

Although only eighty three participants responded to the questionnaire, resulting in a response rate of 25.4%, this modest rate is consistent with response patterns typically observed in survey-based research involving academic populations—particularly when the subject matter pertains to administrative, managerial, or bureaucratic issues, which generally attract lower levels of engagement (Baruch and Holtom, 2008; Fan and Yan, 2010; Harzing et al., 2013).

The demographic data (Table 1) showed a predominance of male researchers, aged 34–39 years, holding a doctoral or postdoctoral degree, which correlated with a higher rate of project submissions, particularly in categories with a greater number of proposals. Male researchers submitted a notably greater number of projects, suggesting a gender disparity in the field of research. In addition, women may take longer to consolidate their academic careers due to additional challenges, such as unequal opportunities, difficulties in balancing professional and personal demands, and less involvement in strategic academic networks (Costa et al., 2025).

Gender disparities in academic careers, potential disadvantages for women researchers (Bol et al., 2022) influencing the organizational context, and the impact of phenomena such as the “Matthew effect,” (Hu, 2020), where male researchers are viewed as more central and important, and the “Matilda effect,” where the work of women is undervalued or their ideas are attributed to male researchers, significantly affect research and innovation dynamics (Ovseiko et al., 2016; Dion et al., 2018). Structural and cultural factors, such as the persistence of gender stereotypes and the lower representation of women in leadership positions and scientific societies, further exacerbate these disparities (Costa et al., 2025). Moreover, gender inequality is reflected not only in the absolute number of researchers but also in the unequal distribution of funding opportunities, leadership roles, and scientific visibility (Bendels et al., 2018; Nittrouer et al., 2018).

As a consequence of inequality, this situation goes beyond mere numerical representation, permeating career advancement, recognition, and access to resources (Morais et al., 2022). On the other hand, the underrepresentation of women was evidenced in different scenarios of *CNPq* fellowship distribution (Oliveira et al., 2021), which is a mark of excellence in the scientific career in Brazil (Costa et al., 2025). Reflecting on these aspects is crucial for analyzing the relationships between demographic characteristics. Participation in graduate programs and *CNPq* fellowships was a statistically significant factor, suggesting that academic involvement and funding are fundamental to research productivity (Picinin et al., 2016; McGill and Settle, 2012).

Table 2 and the Supplementary material 3, 4 demonstrate that the primary reason for submitting projects was to secure funding for their execution. Another reason that was often indicated is related to the need to strengthen academic curricula, which is fueled by the well-documented “publish or perish” culture (Rond and Miller, 2005). In this context, it intensifies competition, affects research quality and innovation (Fanelli, 2010), and reinforces cumulative advantages described by the “Matthew Effect” (Hu, 2020).

These findings highlighted systemic issues within the research funding landscape in Brazil, corroborating other studies that investigated the importance of funding for academic research (Tsugawa et al., 2022). Generally, the higher the success rate of researchers, the more universities and the government will actively promote scientific research (Zheng, 2023). In essence, when investment yields benefits for society, it is natural for research activities to be valued, promoting a virtuous cycle of progress, development, and opportunities (Costa et al., 2025). This dynamic also reflects the well-established understanding that economic growth has an intimate and interactive relationship with scientific innovation, which supports economic growth and drives scientific innovation (Zhang et al., 2012). Therefore, scientific development and technological innovation are interdependent and essential for the sustainable development and growth of society and its productive forces (Costa et al., 2025). In the Brazilian context, the issues include overreliance on competitive calls, bureaucratic hurdles, and insufficient institutional support, all of which reinforce barriers to equitable participation in research. Addressing these challenges requires not only institutional changes but also broader policy interventions to reduce structural vulnerabilities in the research ecosystem.

Ensuring funding from diverse sources is an ongoing necessity to support research activities, identify funding agencies, understand their mechanisms, and form collaborative research teams (Lee, 2016). Furthermore, funding security is an indicator of success and often influences an organization's recruitment and promotion decisions (Hu et al., 2015). Past productivity and scientific collaboration also affect funding security (Ebadi and Schiffauerova, 2015; Hu, 2020; Davies et al., 2022), reinforcing the perception that funding is the primary concern of academic researchers. In this regard, the data stratification by researchers who answered “Yes” to having approved projects revealed an interesting pattern when this approval category was divided into two groups: “1–3” and “4 or more” projects. In addition, researchers who submitted “7 or more” projects had a significantly higher approval rate, with 58.3% obtaining “4 or more” approvals (Figure 1). These data suggested that persistence and experience with the submission process are associated with higher rates of project approval. This pattern indicates that continual practice and learning from previous submissions can effectively increase future project approval rates (Ardehali, 2014).

Our findings reinforced that barriers to research project submission and approval—such as bureaucratic funding calls, short deadlines, and limited institutional support—are not isolated challenges but part of a broader systemic issue that continues to constrain research productivity in Brazil. These barriers were particularly salient among researchers without established collaboration networks or sufficient infrastructure, underscoring how structural inequities in access to resources, exacerbate disparities within the academic community, corroborating previous studies (Tamblyn et al., 2018; Gillespie et al., 2001; Souza et al., 2020) highlights the persistence of these systemic issues. These challenges were documented in the literature (Linton, 2008) highlighting the need for greater clarity and support from funding agencies. However, our results further demonstrate that these barriers are not experienced uniformly; instead, they intersect with gender, career stage, and institutional context, amplifying difficulties for specific groups. This synthesis suggests that addressing research funding challenges requires not only administrative simplification but also targeted institutional policies to reduce inequities and foster inclusive research environments.

Researchers who submitted 4–6 projects reported difficulties in finding institutional partnerships, indicating a stage in which the researcher tries to expand their network but does not yet have a solid collaboration base (Kezar, 2005). The development of a collaborative network is key to project success, as it increases resources and specialized knowledge, thereby expanding the reach and impact of studies (Zhang et al., 2018; Vasilyeva et al., 2021) and enhancing academic production through co-authorships (Davies et al., 2022). Differences between researchers who submitted projects and those who did not reflect varied expertise levels and different understandings of the evaluation criteria (Table 4). This suggests that better communication and guidance regarding these criteria are necessary for early-stage researchers (Bloomfield et al., 2016).

To address these challenges, it is crucial to implement structured grant writing training, mentorship programs involving experienced principal investigators, and institutional initiatives that actively foster collaboration networks. Additionally, providing clear and accessible information about funding opportunities and evaluation processes can empower researchers, particularly in contexts where research is optional but represents a key element of career development (Baan et al., 2020). Furthermore, factors such as the satisfaction derived from mentoring, interest in research activities, and institutional incentives remain central to motivating faculty participation in research (Costa et al., 2025). These recommendations highlight the importance of fostering research engagement as an integral part of academic careers in federal institutes.

Strategies that indicated increased approval rates included adapting the project to the established criteria and demonstrating its relevance and impact (Table 3). These results corroborate the literature, which highlights that effective funding security strategies include developing innovative ideas, choosing appropriate funding agencies, and preparing well-organized proposals (Koppelman and Holloway, 2012). Proposals must provide academic innovation, scientific rigor, and potential impact; meet the criteria of funding agencies; and highlight both the public importance and the empirical basis of the project presented (Proctor et al., 2012). The continual development of these skills is essential in a competitive and dynamic academic research context, as they increase the possibility of funding approval and publication rates, thus contributing to the researcher's career (Edwards et al., 2023; Castillo-Martínez and Ramírez-Montoya, 2021).

The process of submitting and approving a research project involves important lessons for researchers. The results affirmed that only clear research proposals were approved. Another relevant factor is the researcher's expertise, as confirmed by the literature (Hu, 2020), or the research team, as shown by lower means and significant *p*-values (Table 2). Therefore, appropriate methodological planning, resource availability, and compliance with ethical standards are essential; and another relevant point is that researchers with approved projects stated that having resources such as laboratories, equipment, and partnerships or collaborations with other institutions increases approval rates. These results suggested that experience with the evaluation process increases knowledge of what funding agencies expect. This can positively influence future project submissions and approvals. Some studies have reported the importance of knowing the evaluation criteria and financing mechanisms, as well as the formation of interdisciplinary teams (Prendergast et al., 2008; Lee, 2016). The literature also highlights the analysis of funding trends to understand funding priorities (Faisal et al., 2020) and the importance of compatible objectives between researchers and agencies (Tetroe et al., 2008).

The multivariate logistic regression analysis (Tables 3, 4) showed how the different parameters affected project submission and approval. Female sex was associated with a 53% decrease in project submission approval (*OR* = 0.47), which is a barrier in this context. Similarly, less clear projects were 32% less likely to be submitted (*OR* = 0.68), and reduced expertise was associated with a 69% reduction in the likelihood of submission (*OR* = 0.31). Concerning project approval, factors such as the possibility of funding (*OR* = 1.15), strengthening the academic curriculum (*OR*=1.41), and institutional stimulus (*OR*=1.38) had *OR*s that increased by 15%, 41%, and 38%, respectively. These findings indicate that these factors are crucial in securing funding. Conversely, lower resource availability (*OR* = 0.51) and fewer partnerships (*OR* = 0.39) decreased the approval rates by 49% and 61%, respectively, indicating the importance of institutional support and collaboration in the research process. Having children had an adverse effect on both processes, decreasing the chances of submission and approval by 30% (*OR* = 0.70) and 56% (*OR* = 0.44), respectively, suggesting that parental responsibility may limit participation in research.

Although some studies have shown that the gender of the principal investigator affects funding securement, with women receiving lower evaluations than men do, regardless of the quality of the proposed research (Witteman et al., 2019). The literature provides little direct evidence of the specific effect of motherhood or fatherhood on research funding—a topic that warrants further investigation through an in-depth analysis of the impact of having children on securing financing and research publication. These quantitative data are crucial for understanding the effects of each parameter, guiding the development of policies, and reducing the barriers faced by researchers.

### 4.1 Limitations and strengths

This study has several limitations that should be acknowledged. First, the response rate was relatively low, which aligns with patterns commonly observed in survey-based research involving academic populations; this limitation restricts the generalizability of the findings beyond the institutional context analyzed. Second, the impossibility of conducting a non-response bias analysis —due to the anonymity of the responses and the lack of access to complete demographic data—is a common constraint in Brazilian public institutions. Third, focusing on a single institution restricts the external validity of the results, as contextual factors may influence the challenges and perceptions reported. Although the study was conducted in a single institution, the findings highlight systemic patterns and institutional challenges that are potentially shared across similar contexts, especially within the broader *RFEPCT* in Brazil. Future studies could expand this approach to multicenter designs or regional comparisons, thus enhancing external validity and supporting evidence-based policy development at national and international levels.

However, the study presents strengths as it fills a gap in the literature by examining research funding challenges within the underexplored Brazilian context, particularly in federal institutions of professional and technological education. Additionally, the use of robust statistical techniques, such as logistic regression, enhances the reliability of the results by enabling the identification of factors associated with submission and approval outcomes. Lastly, the comprehensive questionnaire captured various variables related to motivations, barriers, and experiences in the research funding process.

## 5 Conclusion

This study evaluated the perceptions, barriers, and challenges faced by *IF Goiano* researchers during the project submission process for approval by funding agencies. The results reveal a complex scenario characterized by gender disparities, the impact of demographic factors and parenthood, and the need for more assertive public policies and institutional strategies to secure equitable funding opportunities. To address these challenges, we suggest the implementation of gender-sensitive evaluation criteria in grant calls, creating mentoring programs for early-career and underrepresented researchers, and offering institutional flexibility through extended deadlines or support mechanisms for those with caregiving responsibilities. Although the lower likelihood of project submissions by women indicates structural inequalities requiring attention, our findings also highlight other critical dimensions—such as career stage, academic rank, and institutional support—that influence access to research funding. The central factors driving submission were the search for financing, curriculum strengthening, and structural challenges, including complex funding calls and short deadlines. These findings not only reflect the unique institutional and structural barriers faced by researchers in Brazilian federal institutes but also empirically reinforce theoretical concerns discussed in the literature, including the Matthew and Matilda effects, the cumulative advantage phenomenon, and the influence of hyper competition and institutional prestige on funding outcomes. Additionally, the observed gender disparities and the lower submission and approval rates among researchers with caregiving responsibilities echo prior evidence that systemic and cultural factors continue to shape access to research opportunities. Our study not only highlighted the importance of implementing inclusive institutional policies and effective support systems but also provide a foundation for future studies, such as employing mixed methods, to further explore funding challenges in Brazilian science. These recommendations align with the international research agenda, which advocates for inclusive science, gender equity, and democratized funding. By drawing on evidence from a Latin American context, this study underscores the importance of policies that incorporate local realities into global scientific strategies.

## Data Availability

Data cannot be shared publicly due to the ethical restrictions imposed by Brazilian legislation (Resolution *CNS* No. 466, dated December 12, 2012). The data are available from the principal investigator of this study (C.C.R.), who is responsible for curating the dataset collected. In the event of a request for access to data, the authors will determine whether to consider the reasonableness of the request in light of the need to protect the opinions and information of the research participants.
