# Supplementary material for: Research funding challenges in Brazil: researchers' perceptions from a public institution of professional education
Source: Front Res Metr Anal. 2025 Sep 22;10:1553928. doi: 10.3389/frma.2025.1553928 (PMC12497820; doi:10.3389/frma.2025.1553928)
Supplement: Supplementary file 1 [file Table_1.pdf]

Supplementary Material S3

Researchers' perspectives on the influence of project submission and outcomes.

**Parameters influencing**	***n* (%)**	**Research projects submission****			***p****
		**1–3**	**4–6**	**≥7**	
Reasons for submitting projects to funding calls***					
Possibility of obtaining funding to conduct the project	57 (91.9)	27 (47.4)	19 (33.3)	11 (19.3)	0.266
Increase the research impact and visibility	29 (46.8)	12 (41.4)	10 (34.5)	7 (24.1)	0.424
Access to additional resources and infrastructure	39 (62.9)	18 (46.2)	13 (33.3)	8 (20.5)	0.279
Strengthening the academic curriculum	30 (48.4)	14 (46.7)	9 (30.0)	7 (23.3)	0.736
Institutional encouragement to submit projects	20 (32.3)	7 (35.0)	7 (35.0)	6 (30.0)	0.298
Difficulties faced when submitting research projects to calls for proposals***					
Complex requirements and criteria for public calls	34 (54.8)	17 (50.0)	12 (35.3)	5 (14.7)	0,504
Limited availability of funding resources	21 (33.9)	9 (42.9)	8 (38.1)	4 (19.0)	0.637
Competition with other researchers	29 (46.8)	14 (48.3)	11 (37.9)	4 (13.8)	0.397
Difficulty in finding institutional partnerships	19 (30.6)	6 (31.6)	11 (57.9)≠	2 (10.5)	**0.008**
Difficulty in finding collaborating researchers	13 (21.0)	6 (46.2)	6 (46.2)	1 (7.7)	0.287
Time and effort required to prepare the project	43 (69.4)	20 (46.5)	16 (37.2)	7 (16.3)	0.223
Reason for not submitting to a public funding call, even if interested					
Reconciling time and teaching activities.	1 (1.6)	1 (100.0)	–	–	
Difficulty in meeting the public calls criteria and requirements	9 (14.5)	8 (88.9)	1 (11.1)	–	
Difficulty in finding partnerships or collaborators for the project	3 (4.8)	1 (33.3)	2 (66.7)	–	
Public funding call outside their expertise area	1 (1.6)	–	–	1 (100.0)	0.113
Lack of knowledge about the public calls available	5 (8.1)	3 (60.0)	1 (20.0)	1 (20.0)	
No time	1 (1.6)	–	–	1 (100.0)	
Time constraints for preparing and submitting projects	42 (67.7)	18 (42.9)	15 (35.7)	9 (21.4)	
Factors contributing to the non-approval of research projects***					
The project proposal was not formulated clearly	6 (30.0)	2 (33.3)	4 (66.7)	–	0.132
Lack of previous experience in the research area	8 (40.0)	6 (75.0)	2 (25.0)	–	0.461
The funding agency considered the project to be of low relevance or impact	7 (35.0)	4 (57.1)	3 (42.9)	–	0.690
Lack of adequate resources or infrastructure for the project to be conducted	4 (20.0)	1 (25.0)	3 (75.0)	–	0.169
Difficulties in writing the proposal (e.g., scientific writing)	2 (10.0)	–	1 (50.0)	1 (50.0)	0.055
The budget project presented was considered inadequate	1 (5.0)	–	1 (100.0)	–	0.376
The project proposal did not include national partnerships	1 (5.0)	1 (100.0)	–	–	0.704
The project proposal did not include international partnerships	3 (15.0)	2 (66.7)	1 (33.3)	–	0.902

**Parameters influencing**	***n* (%)**	**Research projects submission****			***p****
		**1–3**	**4–6**	**≥7**	
Approved projects					
None	20 (32.3)	12 (60.0)	7 (35.0)	1 (5.0)	
1–3	35 (56.5)	19 (54.3)	12 (34.3)	4 (11.4)	**<0.001**
4–9	7 (11.3)	–	–	7 (100.0)≠	
Number of submissions until first funding					
1	11 (26.2)	3 (27.3)	5 (45.5)	3 (27.3)	
2–3	21 (50.0)	13 (61.9)	4 (19.0)	4 (19.0)	0.240
≥ 4	10 (23.8)	3 (30.0)	3 (30.0)	4 (40.0)	
Strategies to increase approval chances***					
Seek institutional or collaborative partnerships	32 (76.2)	12 (37.5)	9 (28.1)	11 (34.4)	0.073
Draw up a clear and well-structured project	31 (73.8)	15 (48.4)	8 (25.8)	8 (25.8)	0.747
Adapt the project to the criteria and objectives of the funding call	34 (81.0)	15 (44.1)	8 (23.5)	11 (32.4)	0.121
Make revisions and adjustments based on previous feedback	25 (59.5)	11 (44.0)	6 (24.0)	8 (32.0)	0.530
Demonstrate the project relevance and potential impact	37 (88.1)	17 (45.9)	10 (27.0)	10 (27.0)	0.828
Main criteria influencing approval***					
Scientific project merit	25 (59.5)	11 (44.0)	7 (28.0)	7 (28.0)	0.949
Project relevance and impact on society	27 (64.3)	12 (44.4)	8 (29.6)	7 (25.9)	0.979
Experience and qualifications of the researchers involved	37 (88.1)	17 (45.9)	9 (24.3)	11 (29.7)	0.175
Project technical and methodological feasibility	26 (61.9)	11 (42.3)	9 (34.6)	6 (23.1)	0.534
Alignment with the objectives and priorities of the funding call	27 (64.3)	11 (40.7)	8 (29.6)	8 (29.6)	0.702
Main challenges faced in getting projects approved***					
Fierce competition with other projects	25 (59.5)	12 (48.0)	5 (20.0)	8 (32.0)	0.288
Budget restrictions and limited availability of resources	26 (61.9)	13 (50.0)	9 (34.6)	4 (15.4)	0.119
The complexity of funding call requirements and criteria	11 (26.2)	5 (45.5)	6 (54.5)≠	–	**0.024**
Difficulty in finding collaborative partnerships	9 (21.4)	4 (44.4)	4 (44.4)	1 (11.1)	0.367
Time and effort required to prepare a proposal	27 (64.3)	11 (40.7)	8 (29.6)	8 (29.6)	0.702
Institutional support needed to increase approval chances***					
Guidance in preparing proposals	17 (40.5)	9 (52.9)	3 (17.6)	5 (29.4)	0.432
Financial resources for counterpart or project costs	28 (66.7)	15 (53.6)	8 (28.6)	5 (17.9)	0.172
Specific training on preparing projects and public calls	20 (47.6)	10 (50.0)	4 (20.0)	6 (30.0)	0.500
Encouraging the formation of partnerships and collaboration networks	25 (59.5)	11 (44.0)	10 (40.0)	4 (16.0)	0.071

Notes: The '*n*' values represent absolute frequencies, whereas the '*%*' values represent relative frequencies. * *p*-value for Pearson's parametric chi-square test (χ^2), and bold indicates a result with a statistically significant difference (α=0.05); ≠ indicates the post hoc test. ** This analysis only includes researchers who submitted projects, totaling 62 participants (100% of the sample considered). *** Participants were able to select more than one option in this question.
