# Supplementary material for: Research funding challenges in Brazil: researchers' perceptions from a public institution of professional education
Source: Front Res Metr Anal. 2025 Sep 22;10:1553928. doi: 10.3389/frma.2025.1553928 (PMC12497820; doi:10.3389/frma.2025.1553928)
Supplement: Supplementary file 2 [file Table_2.pdf]

Supplementary Material S4

Sociodemographic and motivational factors influencing project approval rates.

Sociodemographic and motivational factors	*n* (%)	Research projects approved**		*p**
		No	Yes	
Age group				
28–33 years	10 (16.1)	6 (60.0)	4 (40.0)	**0.018**
34–39 years	18 (29.0)	1 (5.6)	17 (94.4)≠	
40–45 years	17 (27.4)	7 (41.2)	10 (58.8)	
≥46 years	17 (27.4)	6 (35.3)	11 (64.7)	
Gender identification				
Female	21 (33.9)	7 (33.3)	14 (66.7)	0.891
Male	41 (66.1)	13 (31.7)	28 (68.3)	
Ethnicity/skin color				
White	34 (54.8)	9 (26.5)	25 (73.5)	0.283
Brown/black	28 (45.2)	11 (39.3)	17 (60.7)	
Marital status				
With a partner	47 (75.8)	16 (34.0)	31 (66.0)	0.595
Without a partner	15 (24.2)	4 (26.7)	11 (73.3)	
Children				
No	22 (35.5)	7 (31.8)	15 (68.2)	0.951
Yes	40 (64.5)	13 (32.5)	27 (67.5)	
Number of children***				
1 child	16 (40.0)	2 (12.5)	14 (87.5)≠	**0.007**
2 children	18 (45.0)	6 (33.3)	12 (66.7)	
3 children	6 (15.0)	5 (83.3)	1 (16.7)	
Youngest child's age ***				
≤1 year	7 (17.5)	3 (42.9)	4 (57.1)	0.976
2–5 years	9 (22.5)	3 (33.3)	6 (66.7)	
6–10 years	7 (17.5)	2 (28.6)	5 (71.4)	
11–17 years	10 (25.0)	3 (30.0)	7 (70.0)	
≥18 years	7 (17.5)	2 (28.6)	5 (71.4)	
Submitted projects				
1–3 projects	31 (37.3)	12 (38.7)	19 (61.3)	0.141
4–6 projects	19 (22.9)	7 (36.8)	12 (63.2)	
≥7 projects	12 (14.5)	1 (8.3)	11 (91.7)	

**Sociodemographic and motivational factors**	***n* (%)**	**Research projects approved****		***p****
		**No**	**Yes**	
Reasons for submitting projects to funding calls****				
Possibility of obtaining funding to conduct the project	57 (91.9)	16 (28.1)	41 (71.9)	**0.017**
Increase the research impact and visibility	29 (46.8)	7 (24.1)	22 (75.9)	0.200
Access to additional resources and infrastructure	39 (62.9)	11 (28.2)	28 (71.8)	0.374
Strengthening the academic curriculum	30 (48.4)	5 (16.7)	25 (83.3)	**0.011**
Institutional encouragement to submit projects	20 (32.3)	3 (15.0)	17 (85.0)	**0.045**
Difficulties faced when submitting research projects to calls for proposals****				
Complex requirements and criteria for calls for proposals	34 (54.8)	11 (32.4)	23 (67.6)	0.896
Limited availability of funding resources	21 (33.9)	7 (33.3)	14 (66.7)	0.987
Competition with other researchers	29 (46.8)	11 (37.9)	18 (62.1)	0.370
Difficulty in finding institutional partnerships	19 (30.6)	6 (31.6)	13 (68.4)	0.939
Difficulty in finding collaborating researchers	13 (21.0)	5 (38.5)	8 (61.5)	0.590
Time and effort required to prepare the project	43 (69.4)	12 (27.9)	31 (72.1)	0.270
Reason for not submitting to a funding call, even if interested				
Reconciling time and teaching activities.	1 (1.6)	1 (100.0)	–	
Difficulty in meeting the criteria and requirements of calls for proposals	9 (14.5)	5 (55.6)	4 (44.4)	
Difficulty in finding partnerships or collaborators for the project	3 (4.8)	1 (33.3)	2 (66.7)	
Calls for proposals outside the expertise area.	1 (1.6)	–	1 (100.0)	0.344
Lack of knowledge about the calls for proposals available	5 (8.1)	2 (40.0)	3 (60.0)	
No time	1 (1.6)	–	1 (100.0)	
Time constraints for preparing and submitting projects	42 (67.7)	11 (26.2)	31 (73.8)	

Notes: The '*n*' values represent absolute frequencies, whereas the '%' values represent relative frequencies. * *p*-value for Pearson's parametric chi-square test (χ^2), and bold indicates a result with a statistically significant difference (α=0.05); ≠ indicates the post hoc test. ** This analysis only includes researchers who submitted projects, totaling 62 participants (100% of the sample considered). *** This question was answered exclusively by participants who said they had children. **** Participants were able to select more than one option in this question.
