# Supplementary material for: Research funding challenges in Brazil: researchers' perceptions from a public institution of professional education
Source: Front Res Metr Anal. 2025 Sep 22;10:1553928. doi: 10.3389/frma.2025.1553928 (PMC12497820; doi:10.3389/frma.2025.1553928)
Supplement: Supplementary file 3 [file Table_3.pdf]

## Supplementary Material S5

Distribution of approved projects, approval strategies, criteria, and institutional support.

| **Parameters** | ***n* (%)** | **Approved projects**** | | ***p**** |
|---|---|---|---|---|
| | | 1–3 | ≥4 | |
| Number of submissions until first funding | | | | |
| 1 project | 11 (26.2) | 9 (81.8) | 2 (18.2) | |
| 23 projects | 21 (50.0) | 18 (85.7) | 3 (14.3) | 0.912 |
| ≥4 projects | 10 (23.8) | 8 (80.0) | 2 (20.0) | |
| Strategies to increase chances of approval*** | | | | |
| Seek institutional or collaborative partnerships | 32 (76.2) | 25 (78.1) | 7 (21.9) | 0.105 |
| Draw up a clear and well-structured project | 31 (73.8) | 25 (80.6) | 6 (19.4) | 0.433 |
| Adapt the project to the criteria and objectives of the public calls | 34 (81.0) | 27 (79.4) | 7 (20.6) | 0.160 |
| Make revisions and adjustments based on previous feedback | 25 (59.5) | 20 (80.0) | 5 (20.0) | 0.482 |
| Demonstrate the project relevance and potential impact | 37 (88.1) | 30 (81.1) | 7 (18.9) | 0.287 |
| Main criteria influencing approval*** | | | | |
| Scientific project merit | 25 (59.5) | 20 (80.0) | 5 (20.0) | 0.482 |
| Relevance and impact of the project for society | 27 (64.3) | 21 (77.8) | 6 (22.2) | 0.195 |
| Experience and qualifications of the researchers involved | 37 (88.1) | 30 (81.1) | 7 (18.9) | 0.285 |
| Project technical and methodological feasibility | 26 (61.9) | 21 (80.8) | 5 (19.2) | 0.580 |
| Alignment with the objectives and priorities of the public calls | 27 (64.3) | 22 (81.5) | 5 (18.5) | 0.666 |
| Main challenges faced in getting projects approved*** | | | | |
| Strong competition with other projects | 25 (59.5) | 20 (80.0) | 5 (20.0) | 0.482 |
| Budget restrictions and limited availability of resources | 26 (61.9) | 24 (92.3)≠ | 2 (7.7) | **0.047** |
| Requirements complexity and criteria of the public calls | 11 (26.2) | 11 (100.0) | 0 (0.0) | 0.084 |
| Difficulty in finding collaborative partnerships | 9 (21.4) | 9 (100.0) | – | 0.130 |
| Time and effort required to prepare a proposal | 27 (64.3) | 23 (85.2) | 4 (14.8) | 0.666 |
| Institutional support needed to increase chances of approval*** | | | | |
| Guidance in preparing proposals | 17 (40.5) | 14 (82.4) | 3 (17.6) | 0.888 |
| Financial resources for counterpart or project costs | 28 (66.7) | 25 (89.3) | 3 (10.7) | 0.143 |
| Specific training on preparing projects and public calls for proposals | 20 (47.6) | 15 (75.0) | 5 (25.0) | 0.167 |
| Encouraging the formation of partnerships and collaboration networks | 25 (59.5) | 23 (92.0) | 2 (8.0) | 0.068 |

**Notes:** The '*n*' values represent absolute frequencies, whereas the '*%*' values represent relative frequencies. * *p*-value for Pearson's parametric chi-square test ($\chi^2$), and bold indicates a result with a statistically significant difference ($\alpha$=0.05); ≠ indicates the post hoc test. ** This analysis only includes researchers who had approved projects, totaling 42 participants (100% of the sample considered).
