# Supplementary material for: Research funding challenges in Brazil: researchers' perceptions from a public institution of professional education
Source: Front Res Metr Anal. 2025 Sep 22;10:1553928. doi: 10.3389/frma.2025.1553928 (PMC12497820; doi:10.3389/frma.2025.1553928)
Supplement: Supplementary file 4 [file Table_4.pdf]

Supplementary Material S6

Researchers' agreement with funding agency evaluation criteria.

Agreement	$M \pm SD$	Strongly agree *n* (%)	Agree *n* (%)	Neither agree nor disagree *n* (%)	Disagree *n* (%)	Strongly disagree *n* (%)
● Proposal clarity	1.81 ± 0.79	32 (38.6)	37 (44.6)	13 (15.7)	–	1 (1.2)
● Project relevance and originality	1.75 ± 0.81	36 (43.4)	35 (42.2)	10 (12.0)	1 (1.2)	1 (1.2)
● Researcher expertise	1.66 ± 0.65	36 (43.4)	39 (47.0)	8 (9.6)	–	–
● Project methodological design	2.06 ± 0.79	17 (20.5)	48 (57.8)	16 (19.3)	–	2 (2.4)
● Resources availability	2.10 ± 0.86	20 (24.1)	41 (49.4)	17 (20.5)	4 (4.8)	1 (1.2)
● Adherence to ethical guidelines	2.07 ± 0.84	20 (24.1)	42 (50.6)	17 (20.5)	3 (3.6)	1 (1.2)
● Partnerships and collaborations between institutions	1.69 ± 0.66	35 (42.2)	39 (47.0)	9 (10.8)	–	–
● Project funding proportion	1.98 ± 0.73	20 (24.1)	47 (56.6)	15 (18.1)	–	1 (1.2)
● Research impact demonstration	1.75 ± 0.75	33 (39.8)	40 (48.2)	9 (10.8)	–	1 (1.2)
● Project/agency priority compatibility	1.70 ± 0.64	33 (39.8)	42 (50.6)	8 (9.6)	–	–
● Project includes S&T popularization	2.12 ± 0.79	17 (20.5)	42 (50.6)	22 (26.5)	1 (1.2)	1 (1.2)

Notes: The '*n*' values represent absolute frequencies, whereas the '*%*' values represent relative frequencies. The final sample size was N=83. 'M' and 'SD' represent the mean and standard deviation, respectively. S&T is the abbreviation for science and technology. The mean values range from 1 to 5, with 1 indicating strong agreement and 5 indicating strong disagreement.
