# Supplementary material for: Research funding challenges in Brazil: researchers' perceptions from a public institution of professional education
Source: Front Res Metr Anal. 2025 Sep 22;10:1553928. doi: 10.3389/frma.2025.1553928 (PMC12497820; doi:10.3389/frma.2025.1553928)
Supplement: Supplementary file 5 [file Data_Sheet_1.pdf]

PARECER CONSUBSTANCIADO DO CEP

DADOS DO PROJETO DE PESQUISA

Título da Pesquisa: FINANCIAMENTO NA PESQUISA CIENTÍFICA: um estudo dos fatores associados às submissões e aprovações de projetos de pesquisa em agências de fomento pelos pesquisadores do IF Goiano

Pesquisador: CRISTHIAN CHAGAS RIBEIRO

Área Temática:

Versão: 2

CAAE: 67695523.4.0000.0036

Instituição Proponente: INSTITUTO FEDERAL DE EDUCACAO, CIENCIA E TECNOLOGIA GOIANO

Patrocinador Principal: Financiamento Próprio

DADOS DO PARECER

Número do Parecer: 6.144.987

Apresentação do Projeto:

Relata-se: “A presente pesquisa tem como objetivo avaliar os fatores associados às submissões e aprovações de projetos de pesquisa em agências de fomento pelos pesquisadores do Instituto Federal de Educação, Ciência e Tecnologia Goiano (IF Goiano). Este estudo consiste em três fases: revisão narrativa da literatura, estudo de caso de abordagem mista sequencial e desenvolvimento de um produto educacional. A revisão narrativa abordará tópicos teóricos ou contextuais. O estudo de caso usará abordagem mista para explorar fatores associados ao sucesso de aprovações em agências de fomento por pesquisadores do IF Goiano. A fase quantitativa incluirá um questionário eletrônico para todos os pesquisadores selecionados, com a amostra final sendo composta pelos voluntários, com margem de erro de 5% e nível de confiança de 95%. Já na fase qualitativa, nove pesquisadores serão entrevistados individualmente, selecionados aleatoriamente em três grupos: aqueles que nunca submeteram propostas de pesquisa, os que submeteram, mas tiveram propostas rejeitadas e os que submeteram e tiveram propostas aprovadas. A análise dos dados coletados será feita por meio do software SPSS 22.0 para a análise descritiva e inferencial dos dados coletados por meio de questionários online, e por meio da técnica de análise de conteúdo de Bardin para os dados coletados nas entrevistas. O estudo será realizado com a aprovação do Comitê de Ética em Pesquisa. Por fim, o estudo resultará em um produto educacional em formato de videoaula e material didático para contribuir na prospecção e aprovação de pesquisas

submetidas a Editais de fomento pelos pesquisadores do IF Goiano. Os benefícios esperados incluem a identificação de possíveis problemas na submissão e aprovação de projetos, bem como a proposição de sugestões para melhorar o processo de financiamento de pesquisas científicas".

Parecer: Com pendência – O percentual da amostra fora corrigido para 5%. Por tal, deve estar especificado tanto no projeto detalhado (como já consta) mas também identificado nas informações básicas do projeto (neste último são informações antigas (2%) e, por isso, incorretas.

Objetivo da Pesquisa:

Relata-se: "

Objetivo Primário: A presente pesquisa tem como objeto avaliar os fatores associados às submissões e aprovações de projetos de pesquisa em agências de fomento pelos pesquisadores do Instituto Federal de Educação, Ciência e Tecnologia Goiano (IF Goiano).

Objetivo Secundário: 1. Descrever o perfil dos pesquisadores do IF Goiano e sua produção científica no período de 2018 a 2022; 2. Identificar as agências de fomento às quais os pesquisadores do IF Goiano submeteram e/ou aprovaram seus projetos; 3. Identificar os principais fatores associados às submissões de projetos de pesquisa a agências de fomento, pelos pesquisadores do IF Goiano; 4. Identificar os principais fatores associados às aprovações de projetos de pesquisa pelas agências de fomento, submetidos por pesquisadores do IF Goiano; 5. Desenvolver um produto educacional, a partir dos resultados da pesquisa, visando contribuir na prospecção e aprovação de pesquisas submetidas à Editais de fomento.

Avaliação dos Riscos e Benefícios:

3 - Avaliação dos Riscos e Benefícios:

Parecer: "Não houve alteração mediante parecer anterior".

Comentários e Considerações sobre a Pesquisa:

4 - Comentários e Considerações sobre a Pesquisa:

Parecer: "Não houve alteração mediante parecer anterior".

4.2 - Relevância Social e objetivos da pesquisa:
Parecer: "Não houve alteração mediante parecer anterior".

4.3- Metodologia, incluindo local, população e amostra, métodos de coleta:
Relata-se: “
Amostra: Para o cálculo amostral deste estudo, será considerada a população de 327 pesquisadores do IF Goiano que trabalharam com pesquisa em 2022. Com um nível de confiança de 95% e margem de erro de 5%, espera-se um número de 177 participantes respondentes.

Parecer: atende a legislação

4.4- Avaliação do processo de obtenção do TCLE:
Parecer: "Não houve alteração mediante parecer anterior".

4.5- Garantias Éticas aos Participantes da Pesquisa:
Parecer: "Não houve alteração mediante parecer anterior".

4.6- Critérios de Inclusão e Exclusão
Parecer: "Não houve alteração mediante parecer anterior".

4.7- Critérios de Encerramento ou Suspensão da Pesquisa
Parecer: "Não houve alteração mediante parecer anterior".

4.8- Resultados do Estudo
Parecer: "Não houve alteração mediante parecer anterior".

4.9- Divulgação dos Resultados
Parecer: "Não houve alteração mediante parecer anterior".

4.10- Cronograma
Parecer: "Não houve alteração mediante parecer anterior".

Endereço: Rua 88, n°280, Prédio SIASS, andar térreo
Bairro: Setor Sul **CEP:** 74.085-010
UF: GO **Município:** GOIANIA
Telefone: (62)99226-3661 **Fax:** (62)3605-3661 **E-mail:** cep@ifgoiano.edu.br

4.11- Orçamento
Parecer: "Não houve alteração mediante parecer anterior".

4.12- Compatibilidade entre currículos dos pesquisadores e a pesquisa
Parecer: "Não houve alteração mediante parecer anterior".

4.13 - Adequação do protocolo de pesquisa em ambientes virtuais (Carta Circular nº 01/2021)
Parecer: "Não houve alteração mediante parecer anterior".

Considerações sobre os Termos de apresentação obrigatória:
5.1- Folha de rosto:
Parecer: "Não houve alteração mediante parecer anterior".

5.2- TCLE: (Exigência IV.4, IV.5 , IV.6 - Res. 466/12)
5.2.a) justificativa
Parecer: "Não houve alteração mediante parecer anterior".

5.2.d) garantia de liberdade de recusa de participação e/ou retirada da pesquisa sem penalizações;
Parecer: "Não houve alteração mediante parecer anterior".

5.2.e) garantia de sigilo e privacidade;
Parecer: "Não houve alteração mediante parecer anterior".

5.2- TCLE: (Exigência IV.4, IV. 5, IV.6 - Res. 466/12)
objetivos e os procedimentos metodológicos;

Relata-se: “objetivos: “FINANCIAMENTO NA PESQUISA CIENTÍFICA: um estudo dos fatores associados às submissões e aprovações de projetos de pesquisa em agências de fomento pelos pesquisadores do IF Goiano”.
Parecer: Atende a legislação.

Endereço: Rua 88, n°280, Prédio SIASS, andar térreo
Bairro: Setor Sul **CEP:** 74.085-010
UF: GO **Município:** GOIANIA
Telefone: (62)99226-3661 **Fax:** (62)3605-3661 **E-mail:** cep@ifgoiano.edu.br

Relata-se: "procedimentos metodológicos: Para a coleta de dados, serão realizadas entrevistas individuais estruturadas e aplicação de questionário fechado com questões abertas. As entrevistas ocorrerão por meio do Google Meet, permitindo maior flexibilidade e comodidade aos participantes. Além disso, as entrevistas serão gravadas para posterior transcrição e análise dos dados por categorias de conteúdo.
Os questionários, por sua vez, serão aplicados utilizando a plataforma Google Forms, facilitando o preenchimento e a organização das respostas. Após a coleta das informações, os questionários serão tabulados conforme análise estatística para combinação dos dados entre os instrumentos de coleta, garantindo uma análise mais completa e abrangente do objeto de estudo".
Parecer: Atende a legislação.

5.2.b) explicitação dos possíveis desconfortos e riscos decorrentes da participação
Relata-se: "Benefícios esperados: A participação neste estudo visa aprimorar o conhecimento sobre fatores que afetam a submissão e aprovação de projetos de pesquisa, fornecendo informações úteis para pesquisadores e gestores do IF Goiano e nacionalmente. A identificação de estratégias eficazes de captação de recursos auxiliará na elaboração de propostas competitivas e fomentará a discussão sobre desafios e oportunidades no financiamento científico, estimulando a colaboração entre pesquisadores, instituições e agências de fomento. A colaboração dos participantes é crucial para obter resultados significativos e confiáveis que possam impactar positivamente as práticas e políticas de financiamento da pesquisa científica".
Parecer: Atende a legislação.

5.2.c) esclarecimento sob a forma de acompanhamento e assistência aos participantes da pesquisa;
Relata-se: "Conforme a Resolução nº 466/12 do Conselho Nacional de Saúde (CNS), os participantes deste estudo receberão assistência adequada durante e após a pesquisa, conforme detalhado abaixo:
II.3.1 - Assistência imediata: Os participantes da pesquisa terão direito à assistência emergencial e sem ônus de qualquer espécie, caso necessitem durante o estudo. A equipe de pesquisa estará disponível para prestar o suporte necessário em situações emergenciais que possam ocorrer durante a realização do questionário ou entrevista.
II.3.2 - Assistência integral: A assistência integral será oferecida aos participantes para atender complicações e danos decorrentes, direta ou indiretamente, da pesquisa. Embora esta pesquisa

apresente riscos mínimos, a equipe de pesquisa se compromete a prestar apoio aos participantes em caso de eventuais complicações ou danos relacionados à sua participação no estudo.
Para garantir o bem-estar dos participantes, a equipe de pesquisa manterá um canal aberto de comunicação para esclarecer dúvidas, prestar assistência e acompanhar eventuais necessidades dos participantes. Caso seja necessário, encaminharemos os participantes para os serviços de saúde ou suporte adequados, garantindo o atendimento integral.
Parecer: Atende a legislação.

5.2.f) garantia do recebimento do TCLE (vias e não cópias);
Relata-se: "A equipe de pesquisa assegura que todos os participantes tenham acesso a uma cópia do Termo de Consentimento Livre e Esclarecido (TCLE) em formato digital para seus próprios registros. O TCLE será enviado em anexo no e-mail de convite à participação da pesquisa e estará disponível para download no questionário do Google Forms.
Ao iniciar o preenchimento do questionário no Google Forms, o participante encontrará um campo específico para marcar, indicando que leu, compreendeu e concorda com os termos do TCLE. Ao marcar esse campo, será disponibilizado um link para download do TCLE, que o participante poderá baixar e guardar para consulta futura.
Desta forma, cada participante terá sua própria via digital do TCLE, garantindo o acesso às informações sobre a pesquisa e seus direitos como participante. Caso haja dúvidas ou necessidade de esclarecimentos adicionais sobre o TCLE, os participantes podem entrar em contato com a equipe de pesquisa".
Parecer: Atende a legislação.

5.2.g) explicitação da garantia do ressarcimento;
Relata-se: "Considerando que a pesquisa será realizada de forma virtual, a equipe de pesquisa entende que os participantes não devem incorrer em despesas significativas relacionadas ao transporte ou alimentação. No entanto, nos comprometemos a garantir o ressarcimento de quaisquer despesas diretamente relacionadas à participação na pesquisa que venham a ocorrer, conforme estabelecido pela Resolução 466/12 do Conselho Nacional de Saúde (CNS).
Caso haja despesas excepcionais e diretamente vinculadas à participação no estudo, como custos com acesso à internet ou utilização de equipamentos específicos, a equipe de pesquisa se compromete a analisar a possibilidade de ressarcimento. Nesses casos, os participantes deverão apresentar comprovantes das despesas realizadas.

Ressaltamos que o ressarcimento não será considerado como pagamento, gratificação ou incentivo financeiro, mas sim como uma compensação pelas despesas realizadas pelos participantes.
Em caso de dúvidas ou necessidade de esclarecimentos adicionais sobre o ressarcimento, os participantes podem entrar em contato com a equipe de pesquisa.
Parecer: Atende a legislação.

Conclusões ou Pendências e Lista de Inadequações:
Prezado(a) Pesquisador(a),

O CEP IF Goiano aprova seu protocolo de pesquisa. Caso haja alguma modificação, solicitamos que seja inserida uma emenda para avaliação. Ao final da pesquisa, insira o relatório final na plataforma. O prazo para envio de relatório final será de no máximo 60 dias após o término da pesquisa.

Considerações Finais a critério do CEP:

Este parecer foi elaborado baseado nos documentos abaixo relacionados:

Tipo Documento	Arquivo	Postagem	Autor	Situação
Informações Básicas do Projeto	PB_INFORMAÇÕES_BÁSICAS_DO_PROJETO_2091206.pdf	25/05/2023 16:08:02		Aceito
Projeto Detalhado / Brochura Investigador	Projeto_de_Pesquisa_CEP_ProfEPT_Cristhian.pdf	25/05/2023 16:07:31	CRISTHIAN CHAGAS RIBEIRO	Aceito
Cronograma	Cronograma.pdf	25/05/2023 16:06:59	CRISTHIAN CHAGAS RIBEIRO	Aceito
Outros	Resposta_as_Pendencias_Cristhian.docx	19/05/2023 13:22:33	CRISTHIAN CHAGAS RIBEIRO	Aceito
TCLE / Termos de Assentimento / Justificativa de Ausência	TCLE.pdf	19/05/2023 13:19:11	CRISTHIAN CHAGAS RIBEIRO	Aceito
Folha de Rosto	Folha_de_Rosto_Assinada.pdf	03/03/2023 17:17:01	CRISTHIAN CHAGAS RIBEIRO	Aceito

Endereço: Rua 88, n°280, Prédio SIASS, andar térreo
Bairro: Setor Sul **CEP:** 74.085-010
UF: GO **Município:** GOIANIA
Telefone: (62)99226-3661 **Fax:** (62)3605-3661 **E-mail:** cep@ifgoiano.edu.br

Orçamento	Orcamento.pdf	03/03/2023 09:19:47	CRISTHIAN CHAGAS RIBEIRO	Aceito
Outros	Curriculo_Lattes_Cristhian_Chagas_Ribeiro.pdf	03/03/2023 09:16:39	CRISTHIAN CHAGAS RIBEIRO	Aceito
Outros	Curriculo_Lattes_Matias_Noll.pdf	03/03/2023 09:15:51	CRISTHIAN CHAGAS RIBEIRO	Aceito
Declaração de Pesquisadores	Termo_de_Compromisso.pdf	20/02/2023 16:22:13	CRISTHIAN CHAGAS RIBEIRO	Aceito

Situação do Parecer:
Aprovado

Necessita Apreciação da CONEP:
Não

GOIANIA, 27 de Junho de 2023

Paula Medeiros Costa

Assinado por:
Paula Medeiros Costa
(Coordenador(a))
