# Supplementary material for: Research funding challenges in Brazil: researchers' perceptions from a public institution of professional education
Source: Front Res Metr Anal. 2025 Sep 22;10:1553928. doi: 10.3389/frma.2025.1553928 (PMC12497820; doi:10.3389/frma.2025.1553928)
Supplement: Supplementary file 6 [file Data_Sheet_2.pdf]

This document is the English translation of Consubstantiated Opinion No. 6.144.987 issued by the Brazilian CEP/CONEP System for the Research Ethics Project CAAE No. 67695523.4.0000.0036. The confirmation can be verified at the following link: https://plataformabrasil.saude.gov.br/

INFORMED OPINION OF THE RESEARCH ETHICS COMMITTEE (CEP)

RESEARCH PROJECT DATA

Research Title: FINANCING IN SCIENTIFIC RESEARCH: a study of the factors associated with the submission and approval of research projects to funding agencies by IF Goiano researchers

Researcher: CRISTHIAN CHAGAS RIBEIRO

Thematic Area:

Version: 2

CAAE: 67695523.4.0000.0036

Proposing Institution: INSTITUTO FEDERAL DE EDUCAÇÃO, CIÊNCIA E TECNOLOGIA GOIANO

Main Sponsor: Self-financing

OPINION DATA

Opinion Number: 6.144.987

Project Presentation:

It is reported: "This research aims to evaluate the factors associated with submitting and approving research projects to funding agencies by researchers at the Instituto Federal de Educação, Ciência e Tecnologia Goiano (IF Goiano). This study consists of three phases: a narrative literature review, a case study with a mixed sequential approach, and an educational product's development. The narrative review will address theoretical or contextual topics. The case study will use a mixed-methods approach to explore factors associated with successful funding agency approvals by IF Goiano researchers. The quantitative phase will include an electronic questionnaire for all selected researchers, with the final sample consisting of volunteers, with a margin of error of 5% and a 95% confidence level. In the qualitative phase, nine researchers will be interviewed individually and randomly selected into three groups: those who have never submitted research proposals, those who have submitted proposals but had them rejected, and those who have submitted proposals and had them approved. The data collected will be analyzed using SPSS software version 22.0 for the descriptive and inferential analysis of the data collected through online questionnaires and using Bardin's content analysis technique for the data collected in the interviews. The study will be carried out with the approval of the Research Ethics Committee. Finally, the study will result in an educational product in the form of a video lesson and teaching material to contribute to the prospecting and approval of research submitted to funding calls by IF Goiano researchers. The expected benefits include identifying possible problems in submitting and approving projects, as well as suggestions for improving the process of funding scientific research."

Address: Rua 88, n°280, Prédio SIASS, first floor
Neighborhood: South Sector **Zip Code:** 74.085-010
Country: GO **City:** GOIÂNIA
Phone: (62) 99226-3661 **Fax:** (62) 3605-3661 **E-mail:** cep@ifgoiano.edu.br

Opinion: Pending – The sample percentage has been corrected to 5%. It should, therefore, be specified both in the detailed project (as it already is) and identified in the basic project information (the latter is old information (2%) and, therefore, incorrect).

Research Objective:

It reports:

"Primary Objective: The purpose of this research is to evaluate the factors associated with the submission and approval of research projects to funding agencies by researchers at the Instituto Federal de Educação, Ciência e Tecnologia Goiano (IF Goiano)."

"Secondary Objective: 1. Describe the profile of IF Goiano researchers and their scientific output from 2018 to 2022; 2. Identify the funding agencies to which IF Goiano researchers have submitted and/or approved their projects; 3. Identify the main factors associated with research project submissions to funding agencies by IF Goiano researchers; 4. To identify the main factors associated with approving research projects by funding agencies submitted by IF Goiano researchers; 5. To develop an educational product, based on the research results, aimed at contributing to the prospecting and approval of research submitted to funding calls."

Assessment of Risks and Benefits:

3 - Assessment of Risks and Benefits:

Opinion: "There was no change to the previous opinion."

Comments and Considerations on the Research:

4 - Comments and Considerations on the Research:

Opinion: "There was no change to the previous opinion."

4.2 - Social relevance and research objectives:

Opinion: "There was no change to the previous opinion."

4.3 - Methodology, including location, population and sample, and collection methods:

It is reported: "Sample: For the sample calculation of this study, the population of 327 IF Goiano researchers who worked with research in 2022 will be considered. With a confidence level of 95% and a margin of error of 5%, 177 participants are expected to respond".

Opinion: Complies with legislation.

4.4 - Evaluation of the process of obtaining the ICF:

Opinion: "There was no change to the previous opinion."

4.5 - Ethical Guarantees for Research Participants:

Opinion: "There was no change to the previous opinion."

4.6 - Inclusion and Exclusion Criteria:

Opinion: "There was no change to the previous opinion."

4.7 - Criteria for Termination or Suspension of Research:

Opinion: "There was no change to the previous opinion."

4.8 - Study results:

Opinion: "There was no change to the previous opinion."

4.9 - Dissemination of Results:

Opinion: "There was no change to the previous opinion."

4.10 - Timetable:

Opinion: "There was no change to the previous opinion."

4.11 - Budget:

Opinion: "There was no change to the previous opinion."

4.12 - Compatibility between researchers' CVs and the research:

Opinion: "There was no change to the previous opinion."

4.13 - Adequacy of the research protocol in virtual environments (Circular Letter no. 01/2021):

Opinion: "There was no change to the previous opinion."

Considerations on the Terms of Mandatory Presentation:

5.1 - Title page:

Opinion: "There was no change to the previous opinion."

5.2 - ICF (Requirement IV.4, IV.5, IV.6 - Res. 466/12):

5.2.a) justification:

Opinion: "There was no change to the previous opinion."

5.2.d) guarantee of freedom to refuse participation and/or withdraw from research without penalty:

Opinion: "There was no change to the previous opinion."

5.2.e) Guarantee of confidentiality and privacy:

Opinion: "There was no change to the previous opinion."

5.2 - ICF (Requirements IV.4, IV.5, IV.6 - Res. 466/12) objectives and methodological procedures:

Reported: Objectives: "FUNDING IN SCIENTIFIC RESEARCH: a study of the factors associated with the submission and approval of research projects to funding agencies by IF Goiano researchers."

Opinion: Complies with legislation.

Report: "Methodological procedures: For data collection, individual structured interviews will be conducted, and a closed questionnaire with open questions will be applied. The interviews will take place via Google Meet, allowing greater flexibility and convenience for the participants. The interviews will also be recorded for later transcription and data analysis using content categories.

The questionnaires, in turn, will be applied using the Google Forms platform, making it easier to fill in and organize the answers. After collecting the information, the questionnaires will be tabulated according to statistical analysis to combine the data between the collection instruments, ensuring a more complete and comprehensive analysis of the object of study."

Opinion: Complies with legislation.

5.2.b) Explanation of possible discomforts and risks arising from participation:

It is reported: "Expected benefits: Participation in this study aims to improve knowledge about factors affecting the submission and approval of research projects, providing useful information for researchers and managers at IF Goiano and nationally. The identification of effective fundraising strategies will assist in preparing competitive proposals and foster discussion about challenges and opportunities in scientific funding, stimulating collaboration between researchers, institutions, and funding agencies. The collaboration of participants is crucial to obtain meaningful and reliable results that can positively impact scientific research funding practices and policies."

Opinion: Complies with legislation.

5.2.c) Clarification on how research participants will be monitored and assisted:

It is reported: "In accordance with Resolution 466/12 of the National Health Council (CNS), the participants in this study will receive adequate assistance during and after the research, as detailed below:

II.3.1 - Immediate assistance: Research participants will have the right to emergency assistance, free of charge of any kind, should they need it during the study. The research team will be available to provide the necessary support in emergency situations that may occur during the questionnaire or interview.

II.3.2 - Comprehensive care: Comprehensive care will be offered to participants to deal with complications and damage arising directly or indirectly from the research. Although this research presents minimal risks, the research team supports participants in case of any complications or damage related to their participation in the study.

To ensure the well-being of the participants, the research team will maintain an open communication channel to answer questions, provide assistance, and monitor their needs. If necessary, we will refer participants to the appropriate health or support services, guaranteeing comprehensive care."

Opinion: Complies with legislation.

5.2.f) Guarantee of receipt of the ICF (copies, not copies):

It is reported: "The research team ensures that all participants have access to a copy of the Informed Consent Form (ICF) in digital format for their records. The ICF will be sent as an attachment to the e-mail inviting participants to take part in the research and will be available to download from the Google Forms questionnaire.

When starting to fill in the questionnaire on Google Forms, the participant will find a specific field to check, indicating that they have read, understood, and agree to the terms of the ICF. By checking this box, a link will be provided to download the ICF, which the participant can download and keep for future reference.

In this way, each participant will have a digital copy of the ICF, guaranteeing access to information about the research and their rights as a participant. If there are any doubts or need further Clarification about the ICF, participants can contact the research team."

Opinion: Complies with legislation.

5.2.g) Explanation of the guarantee of reimbursement:

It is reported, "Considering that the research will be conducted virtually, the research team understands that participants should not incur significant expenses related to transportation or food.

However, we undertake to guarantee reimbursement of any expenses directly related to participation in related to participation in the research, as established by Resolution 466/12 of the National Health Council (CNS).

In the event of exceptional expenses directly linked to participation in the study, such as internet access costs or the use of specific equipment, the research team will analyze the possibility of reimbursement. In such cases, participants must provide proof of the expenses incurred.

We emphasize that reimbursement will not be considered payment, gratuity, or financial incentive but rather compensation for the expenses incurred by the participants. If you have any questions or need further Clarification about reimbursement, please contact the research team.

Opinion: Complies with legislation.

Conclusions or Pending Issues and List of Inadequacies:

Dear Researcher,

CEP of the IF Goiano approves your research protocol. If there are any changes, please insert an amendment for evaluation. Enter the final report on the platform at the end of the research. The deadline for submitting the final report is a maximum of 60 days after the end of the study.

Final considerations at the discretion of the CEP:

This opinion was drawn up based on the documents listed below:

Type of Document	File	Posting	Author	Status
Basic Project Information	PB_INFORMAÇÕES_BÁSICAS_DO_PROJETO_2091206.pdf	25/05/2023 16:08:02		Accepted
Detailed Project / Researcher Brochure	Projeto_de_Pesquisa_CEP_ProfEPT_Cristhian.pdf	25/05/2023 16:07:31	CRISTHIAN CHAGAS RIBEIRO	Accepted
Schedule	Cronograma.pdf	25/05/2023 16:06:59	CRISTHIAN CHAGAS RIBEIRO	Accepted
Other	Response_as_Pendencias_Cristhian.docx	19/05/2023 13:22:33	CRISTHIAN CHAGAS RIBEIRO	Accepted
ICF / Terms of Assent / Justification for Absence	TCLE.pdf	19/05/2023 13:19:11	CRISTHIAN CHAGAS RIBEIRO	Accepted
Cover Sheet	Folha_de_Rosto_Assinada.pdf	03/03/2023 17:17:01	CRISTHIAN CHAGAS RIBEIRO	Accepted

Budget	Orcamento.pdf	03/03/2023 09:19:47	CRISTHIAN CHAGAS RIBEIRO	Accepted
Others	Curriculo_Lattes_Cristhian_Chagas_Ribeiro.pdf	03/03/2023 09:16:39	CRISTHIAN CHAGAS RIBEIRO	Accepted
Others	Curriculo_Lattes_Matias_Noll.pdf	03/03/2023 09:15:51	CRISTHIAN CHAGAS RIBEIRO	Accepted
Researchers' Declaration	Termo_de_Compromisso.pdf	20/02/2023 16:22:13	CRISTHIAN CHAGAS RIBEIRO	Accepted

Status of Opinion:

Approved

Needs CONEP appraisal:

No

GOIANIA, June 27, 2023

Signed by:

Paula Medeiros Costa

(Coordinator)
