# Supplementary material for: Research funding challenges in Brazil: researchers' perceptions from a public institution of professional education
Source: Front Res Metr Anal. 2025 Sep 22;10:1553928. doi: 10.3389/frma.2025.1553928 (PMC12497820; doi:10.3389/frma.2025.1553928)
Supplement: Supplementary file 7 [file Data_Sheet_3.pdf]

CONFIRMAR APROVAÇÃO PELO CAAE OU PARECER

Informe o número do CAAE ou do Parecer:

Número do CAAE: 67695523.4.0000.0036

Número do Parecer: 6144987

Pesquisar

Esta consulta retorna somente pareceres aprovados. Caso não apresente nenhum resultado, o número do parecer informado não é válido ou não corresponde a um parecer aprovado.

DETALHAMENTO

Título do Projeto de Pesquisa:

FINANCIAMENTO NA PESQUISA CIENTÍFICA: um estudo dos fatores associados às submissões e aprovações de projetos de pesquisa em agências de fomento pelos pesquisadores do IF Goiano

Número do CAAE: 67695523.4.0000.0036

Número do Parecer: 6144987

Quem Assinou o Parecer: Paula Medeiros Costa

Pesquisador Responsável: CRISTHIAN CHAGAS RIBEIRO

Data Início do Cronograma: 17/02/2023

Data Fim do Cronograma: 29/09/2023

Contato Público: CRISTHIAN CHAGAS RIBEIRO

Voltar

This confirmation can be checked at the following link:
https://plataformabrasil.saude.gov.br/

Then click on the option *'Confirmar Aprovação pelo CAAE ou Parecer'*

Translation from Brazilian Portuguese to American English

You are in: Public > Confirm Approval by CAAE or Opinion

CONFIRM APPROVAL BY CAAE OR OPINION

Enter the CAAE or Opinion number:

CAAE number:
67695523.4.0000.0036

Opinion number:
6144987

This query returns only approved opinions. If there are no results, the opinion number entered is not valid or does not correspond to an approved opinion.

DETAIL

Title of the research project:
SCIENTIFIC RESEARCH FUNDING: a study of the factors associated with the submission and approval of research projects to funding agencies by IF Goiano researchers

CAAE number:
67695523.4.0000.0036

Opinion number:
6144987

Who Signed the Opinion:
Paula Medeiros Costa

Researcher in Charge:
CRISTHIAN CHAGAS RIBEIRO

Date Start of Schedule:
17/02/2023

End Date of Schedule:
29/09/2023

Public Contact:
CRISTHIAN CHAGAS RIBEIRO
