# Supplementary material for: Research funding challenges in Brazil: researchers' perceptions from a public institution of professional education
Source: Front Res Metr Anal. 2025 Sep 22;10:1553928. doi: 10.3389/frma.2025.1553928 (PMC12497820; doi:10.3389/frma.2025.1553928)
Supplement: Supplementary file 8 [file Data_Sheet_4.pdf]

Supplementary Material S1
Survey questionnaire – Brazilian Portuguese original version

Perfil do Pesquisador do IF Goiano

Prezado Pesquisador,

Somos pesquisadores do Instituto Federal Goiano (IF Goiano) e estamos realizando uma pesquisa com temática o FINANCIAMENTO NA PESQUISA CIENTÍFICA E QUALIDADE DE VIDA DO PESQUISADOR. Os resultados podem fornecer insights úteis para orientar melhores práticas para pesquisadores, agências de fomento e instituições de pesquisa no futuro. Contamos com sua preciosa participação.

Bloco A – DECISÃO DO CONVIDADO

O tempo estimado para responder integralmente o questionário é cerca de 15 minutos. Desde já, agradecemos imensamente a sua colaboração!

E-mail: ____

Termo de Consentimento Livre e Esclarecido

Este estudo, que foi aprovado pelo Comitê de Ética em Pesquisa (Protocolo CAAE nº 67695523.4.0000.0036 e Parecer nº 6.144.987). Antes de começar, por favor, acesse o TCLE para decidir sobre sua participação voluntária.

O TCLE está em conformidade com o item IV.5, alínea "a" da Resolução CNS nº 466/2012, cumprindo as exigências do item IV.3. Se você optar por participar, ao selecionar "CONCORDO" e enviar suas respostas, você dará seu consentimento informado e voluntário. Sua participação é essencial para este estudo, mas você tem liberdade para desistir a qualquer momento, sem nenhuma penalidade. Ao selecionar "DISCORDO", não haverá nenhuma penalidade para você.

Após ter lido e compreendido o Termo de Consentimento Livre e Esclarecido (TCLE), você aceita e concorda em participar deste estudo?

◯ **Concordo** ◯ **Discordo**

A indicação do link do CV Lattes proporcionará a extração, por nossa parte, de toda a sua produção científica, diminuindo a quantidade de perguntas deste questionário e, consequentemente, otimizando o seu tempo de resposta.

Informe o link para o seu CV Lattes (opcional): ____

Bloco B – DADOS SOCIODEMOGRÁFICOS

b1. Qual é a sua idade? __________ (em anos)

b2. Você se identifica com o gênero:

- ◯ Feminino
- ◯ Masculino
- ◯ Não binário
- ◯ Prefiro não responder
- ◯ Outro: ____

b3. Qual a sua cor de pele/etnia?

◯ Preta ◯ Parda ◯ Branca ◯ Amarela ◯ Indígena

b4. Qual o seu estado civil?

- ◯ Solteiro
- ◯ Casada ou com união estável
- ◯ Divorciado ou separado
- ◯ Viúvo

Bloco C – SOBRE O TRABALHO

c1. Qual o seu local de trabalho? (Indique a unidade principal do IF Goiano em que você está lotado ou com a qual colabora. Se você atua em mais de uma unidade, selecione apenas a que considera sua principal).

- ◯ Reitoria – Goiânia
- ◯ Campus Avançado de Catalão
- ◯ Campus Avançado de Hidrolândia
- ◯ Campus Avançado de Ipamerí
- ◯ Campus Campos Belos
- ◯ Campus Ceres
- ◯ Campus Cristalina
- ◯ Campus Iporá
- ◯ Campus Trindade
- ◯ Campus Morrinhos
- ◯ Campus Rio Verde
- ◯ Campus Urutaí
- ◯ Polo de Inovação

c2. Identificação Profissional

◯ Docente ◯ Técnico Administrativo

c3. Qual é a sua carga horária de trabalho no IF Goiano?

- ◯ Até 20 horas semanais
- ◯ Mais de 20 horas até 40 horas semanais
- ◯ Mais de 40 horas semanais

c4. Qual é o seu vínculo com o IF Goiano?

- ◯ Servidor efetivo (concursado)
- ◯ Servidor temporário (contrato temporário)
- ◯ Colaborador externo
- ◯ Outro: ____

c5. Qual é o seu tempo de serviço no IF Goiano? __________ (em anos)

c6. Qual é a sua faixa de remuneração mensal bruta?

- ◯ Até R$ 2.999,99
- ◯ R$ 3.000,00 a R$ 5.999,99
- ◯ R$ 6.000,00 a R$ 8.999,99
- ◯ R$ 9.000,00 a R$ 11.999,99
- ◯ R$ 12.000,00 a R$ 14.999,99
- ◯ R$ 15.000,00 a R$ 17.999,99
- ◯ R$ 18.000,00 ou mais
- ◯ Prefiro não responder

c7. Pensando na sua remuneração mensal, você diria que está:

- ◯ Muito satisfeito
- ◯ Satisfeito
- ◯ Nem satisfeito nem insatisfeito
- ◯ Insatisfeito
- ◯ Muito insatisfeito

c8. Qual é o seu mais alto grau de instrução alcançado até o momento?

- ◯ Graduação
- ◯ Pós-graduação lato sensu (especialização)
- ◯ Mestrado
- ◯ Doutorado
- ◯ Pós-doutorado

c9. Você cursou algum grau em instrução fora do Brasil (integral ou parcial)?

- ◯ Não
- ◯ Sim, graduação
- ◯ Sim, pós-graduação lato sensu (especialização)
- ◯ Sim, mestrado
- ◯ Sim, doutorado
- ◯ Sim, pós-doutorado
- ◯ Outro: ____

c10. Além da sua atividade de docente ou técnico administrativo, você ocupa algum outro cargo ou função no IF Goiano?

◯ Sim ◯ Não (Pular para a pergunta [c12])

c11. Especifique as outras funções que você desempenha no IF Goiano:

- ◯ Reitor
- ◯ Pró-Reitoria ou Diretoria-Geral
- ◯ Diretoria
- ◯ Coordenação-Geral, Gerência ou Diretoria-Adjunta
- ◯ Coordenação
- ◯ Chefia de Unidade
- ◯ Chefia de Seção
- ◯ Chefia de Divisão
- ◯ Chefia de Setor
- ◯ Outro: ____

c12. Além do IF Goiano, você atua em outras instituições de ensino superior ou em outra rede de ensino?

◯ Sim ◯ Não (Pular para a pergunta [c14])

c13. Qual é a carga horária semanal de trabalho que você dedica em outras instituições de ensino superior ou em outra rede de ensino além do IF Goiano?

◯ Até 20 horas semanais
◯ Mais de 20 horas até 40 horas semanais
◯ Mais de 40 horas semanais

c14. Nos últimos 5 anos, quanto tempo, em média, você gastou por semana em atividades de ensino?

◯ Menos de 5 h ◯ 5–10 h ◯ 11–20 h ◯ 21–30 h ◯ Mais de 30 h

c15. Nos últimos 5 anos, quanto tempo, em média, você gastou por semana em atividades de pesquisa?

◯ Menos de 5 h ◯ 5–10 h ◯ 11–20 h ◯ 21–30 h ◯ Mais de 30 h

c16. Nos últimos 5 anos, quanto tempo, em média, você gastou por semana em atividades de extensão?

◯ Menos de 5 h ◯ 5–10 h ◯ 11–20 h ◯ 21–30 h ◯ Mais de 30 h

Pensando na sua rotina de trabalho, por favor, leia as afirmativas abaixo e indique se você concorda muito, concorda, não concorda nem discorda, discorda ou discorda muito de cada uma delas.

c17. Passo a maior parte do meu tempo ocupado com tarefas administrativas.

◯ Concordo muito
◯ Concordo
◯ Não concordo nem discordo
◯ Discordo
◯ Discordo muito

c18. Tenho conseguido progredir rapidamente na minha carreira.

◯ Concordo muito
◯ Concordo
◯ Não concordo nem discordo
◯ Discordo
◯ Discordo muito

c19. Tenho tempo suficiente para trabalhar em meus artigos.

◯ Concordo muito
◯ Concordo
◯ Não concordo nem discordo
◯ Discordo
◯ Discordo muito

c20. Não vale a pena seguir a carreira de pesquisador no Brasil.

◯ Concordo muito
◯ Concordo
◯ Não concordo nem discordo
◯ Discordo
◯ Discordo muito

c21. Tenho facilidade em obter financiamento para minhas pesquisas.

◯ Concordo muito
◯ Concordo
◯ Não concordo nem discordo
◯ Discordo
◯ Discordo muito

c22. Minha carga horária em atividades de docência é muito alta.

◯ Concordo muito
◯ Concordo
◯ Não concordo nem discordo
◯ Discordo
◯ Discordo muito

c23. Minha produção intelectual é bastante prejudicada por outras tarefas.

◯ Concordo muito
◯ Concordo
◯ Não concordo nem discordo
◯ Discordo
◯ Discordo muito

c24. Me sinto bastante motivado para trabalhar.

◯ Concordo muito
◯ Concordo
◯ Não concordo nem discordo
◯ Discordo
◯ Discordo muito

c25. Você atua como professor em algum programa de pós-graduação stricto sensu?

◯ Sim ◯ Não

c26. Há quantos tempo você atua como pesquisador? ___________ (em anos)

c27. Você participa de algum projeto ou grupo de pesquisa?

◯ Sim ◯ Não (Pular para a pergunta [c28])

c28. Qual o seu papel nos grupos de pesquisa? (Marque a opção que representa a função mais atuante que você exerce nos grupos de pesquisa em que participa).

- ◯ Líder (responsável por coordenar e planejar os trabalhos de pesquisa do grupo)
- ◯ Pesquisador (servidor graduado ou pós-graduado envolvido com o desenvolvimento de projetos e produção científica, tecnológica e artística do grupo de pesquisa)
- ◯ Técnico (servidor responsável pelo apoio técnico para a realização de projetos de pesquisa e inovação e para a produção científica, tecnológica e artística do grupo)
- ◯ Estudante (discente que participa ativamente da produção científica e tecnológica vinculada às linhas de pesquisa sob a orientação de pesquisadores do grupo de pesquisa)
- ◯ Membro externo (pesquisador, técnico ou estudante com vínculo ativo em outra Instituição de Ensino que contribua no desenvolvimento de projetos e produção científica, tecnológica e artística do grupo de pesquisa)

c29. No IF Goiano, você se sente incentivado a realizar pesquisas?

- ◯ Sim
- ◯ Parcialmente (algumas vezes)
- ◯ Não
- ◯ Não sei ou prefiro não responder

c30. Nesta instituição, você se sente incentivado a publicar seus resultados de pesquisa?

- ◯ Sim
- ◯ Parcialmente (algumas vezes)
- ◯ Não
- ◯ Não sei ou prefiro não responder

c31. Você tem o hábito de fazer pesquisas para aprimorar seu desempenho na ação docente ou no trabalho como técnico administrativo?

- ◯ Sim, frequentemente
- ◯ Sim, algumas vezes
- ◯ Raramente
- ◯ Não, nunca

c32. Na sua percepção, o IF Goiano oferece condições adequadas para que os professores e técnicos administrativos desenvolvam competências e atuem na produção do conhecimento?

- ◯ Sim
- ◯ Parcialmente (algumas vezes)
- ◯ Não
- ◯ Não sei ou prefiro não responder

Bloco D – FATORES RELACIONADOS À SUBMISSÃO DE PROJETOS

d1. Nos últimos 5 anos, quantos projetos de pesquisa você submeteu a editais de financiamento?

- ◯ Nenhum (Pular para a pergunta [f1])
- ◯ De 1 a 3
- ◯ De 4 a 6
- ◯ De 7 a 9
- ◯ De 10 a 12
- ◯ De 13 a 15
- ◯ 16 ou mais

d2. Quais são os principais motivos que influenciam a sua decisão de submeter projetos de pesquisa a editais de financiamento? (Marque todas as opções aplicáveis).

- ◯ Possibilidade de obter financiamento para executar o projeto
- ◯ Ampliar o impacto e a visibilidade da pesquisa
- ◯ Acesso a recursos e infraestrutura adicionais
- ◯ Fortalecimento do currículo acadêmico
- ◯ Estímulo institucional para submissão de projetos
- ◯ Outro: ______________________________________

d3. Quais são as principais dificuldades enfrentados ao submeter projetos de pesquisa a editais de financiamento? (Marque todas as opções aplicáveis).

- ◯ Requisitos e critérios complexos dos editais
- ◯ Disponibilidade limitada de recursos de financiamento
- ◯ Concorrência com outros pesquisadores
- ◯ Dificuldade em encontrar parcerias institucionais
- ◯ Dificuldade em encontrar parcerias de pesquisadores colaboradores
- ◯ Tempo e esforço exigidos na elaboração do projeto
- ◯ Outro: ______________________________________

d4. Quando você deixa de submeter a algum edital, mesmo tendo o interesse, qual o motivo principal para a não submissão?

- ◯ Falta de conhecimento sobre os editais de fomento disponíveis
- ◯ Dificuldade em encontrar parcerias ou colaboradores para o projeto
- ◯ Restrições de tempo para elaboração e submissão de projetos
- ◯ Dificuldade em atender aos critérios e requisitos dos editais
- ◯ Falta de orientação e suporte institucional para submissão de projetos
- ◯ Outro: ______________________________________

Bloco E – FATORES RELACIONADOS À APROVAÇÃO DE PROJETOS

e1. Nos últimos 5 anos, quantos projetos de pesquisa submetidos por você foram aprovados em editais de financiamento?

- ◯ Nenhum (Pular para a pergunta [g1])
- ◯ De 1 a 3
- ◯ De 4 a 6
- ◯ De 7 a 9
- ◯ 10 ou mais

e2. Após quantas submissões a editais de fomento você conseguiu financiar sua primeira pesquisa?

- ◯ Na primeira vez
- ◯ De 2 a 3
- ◯ De 4 a 5
- ◯ De 6 a 7
- ◯ De 8 a 9
- ◯ 10 ou mais

e3. Quais estratégias você utiliza para aumentar as chances de aprovação dos seus projetos de pesquisa em editais de financiamento? (Marque todas as opções aplicáveis).

- ◯ Buscar parcerias institucionais ou colaborativas
- ◯ Elaborar um projeto claro e bem estruturado
- ◯ Adaptar o projeto aos critérios e objetivos do edital
- ◯ Realizar revisões e ajustes com base em feedbacks anteriores
- ◯ Demonstrar a relevância e impacto potencial do projeto
- ◯ Outro: ____

e4. Na sua percepção, quais são os principais critérios que influenciam a aprovação de projetos em editais de financiamento? (Marque todas as opções aplicáveis).

- ◯ Mérito científico do projeto
- ◯ Relevância e impacto do projeto para a sociedade
- ◯ Experiência e qualificação dos pesquisadores envolvidos
- ◯ Viabilidade técnica e metodológica do projeto
- ◯ Alinhamento com os objetivos e prioridades do edital
- ◯ Outro: ____

e5. Na sua opinião, quais são os principais desafios enfrentados na obtenção de aprovação de projetos em editais de financiamento? (Marque todas as opções aplicáveis).

- ◯ Concorrência acirrada com outros projetos
- ◯ Restrições orçamentárias e disponibilidade limitada de recursos
- ◯ Complexidade dos requisitos e critérios dos editais
- ◯ Dificuldade em encontrar parcerias colaborativas
- ◯ Tempo e esforço exigidos na elaboração da proposta
- ◯ Outro: ____

e6. Que tipo de apoio institucional você considera necessário para aumentar as chances de aprovação de projetos em editais de financiamento? (Marque todas as opções aplicáveis).

- ◯ Orientação na elaboração de propostas
- ◯ Recursos financeiros para contrapartida ou custeio do projeto
- ◯ Capacitações específicas sobre elaboração de projetos e editais
- ◯ Estímulo à formação de parcerias e redes de colaboração
- ◯ Outro: ____

Bloco F – FATORES RELACIONADOS À NÃO SUBMISSÃO DE PROJETOS

f1. Nos últimos 5 anos, quais instituições foram responsáveis por financiar seus projetos de pesquisa? (Liste cada das instituições separadas por vírgula).

____

____

____

f2. Nos últimos 5 anos, qual foi o montante total de financiamento que você obteve para suas pesquisas? (Escreva no seguinte formato: R$ 70.000,00) ____________________

f3. Qual o motivo principal pelo qual você ainda não submeteu algum projeto de pesquisa a editais de fomento? (Depois, pular para a pergunta [h1])

- ◯ Falta de conhecimento sobre os editais de fomento disponíveis
- ◯ Dificuldade em encontrar parcerias ou colaboradores para o projeto
- ◯ Restrições de tempo para elaboração e submissão de projetos
- ◯ Dificuldade em atender aos critérios e requisitos dos editais
- ◯ Falta de orientação e suporte institucional para submissão de projetos
- ◯ Outro: ______________________________

Bloco G – FATORES RELACIONADOS À NÃO APROVAÇÃO DE PROJETOS

g1. Quais fatores você acredita que contribuíram para a não aprovação dos seus projetos de pesquisa? (Selecione todas as opções que se aplicam).

- ◯ A proposta do projeto não estava claramente formulada
- ◯ Falta de experiência prévia na área de pesquisa
- ◯ O projeto era considerado de baixa relevância ou impacto pela agência de fomento
- ◯ Falta de recursos ou infraestrutura adequados para executar o projeto
- ◯ Dificuldades na redação da proposta (por exemplo, escrita científica)
- ◯ O orçamento apresentado para o projeto foi considerado inadequado
- ◯ A proposta do projeto não contemplava parcerias nacionais
- ◯ A proposta do projeto não contemplava parcerias internacionais
- ◯ Outro: ______________________________

Bloco H – FATORES RELACIONADOS À SUBMISSÃO E APROVAÇÃO

A partir de sua experiência sobre submissões e aprovações de projetos de pesquisa em agências de fomento, por favor, indique o quanto você concorda com as seguintes afirmativas:

h1. A clareza da proposta de pesquisa é um fator crítico para a aprovação de projetos de pesquisa.

- ◯ Concordo muito
- ◯ Concordo
- ◯ Não concordo nem discordo
- ◯ Discordo
- ◯ Discordo muito

h2. A relevância e originalidade do projeto de pesquisa são essenciais para a sua aprovação.

◯ Concordo muito
◯ Concordo
◯ Não concordo nem discordo
◯ Discordo
◯ Discordo muito

h3. A expertise do pesquisador ou equipe de pesquisa é um fator significativo na aprovação de projetos de pesquisa.

◯ Concordo muito
◯ Concordo
◯ Não concordo nem discordo
◯ Discordo
◯ Discordo muito

O design metodológico do projeto de pesquisa influencia fortemente sua aceitação.

h4. A disponibilidade de recursos (por exemplo, instalações de laboratório, equipamentos etc.) no local de pesquisa impacta a aprovação do projeto de pesquisa.

◯ Concordo muito
◯ Concordo
◯ Não concordo nem discordo
◯ Discordo
◯ Discordo muito

h5. A aderência do projeto a diretrizes éticas é um fator importante para a aprovação de um projeto de pesquisa.

◯ Concordo muito
◯ Concordo
◯ Não concordo nem discordo
◯ Discordo
◯ Discordo muito

h6. A existência de parcerias ou colaborações com outras instituições melhora a probabilidade de aprovação de um projeto de pesquisa.

◯ Concordo muito
◯ Concordo
◯ Não concordo nem discordo
◯ Discordo
◯ Discordo muito

h7. A proporção do orçamento do projeto para a execução da pesquisa é um fator importante para a aprovação do projeto de pesquisa.

◯ Concordo muito
◯ Concordo
◯ Não concordo nem discordo
◯ Discordo
◯ Discordo muito

h8. A demonstração de impacto significativo da pesquisa proposta (impacto social, econômico, ambiental etc.) afeta a aprovação do projeto de pesquisa.

◯ Concordo muito
◯ Concordo
◯ Não concordo nem discordo
◯ Discordo
◯ Discordo muito

h9. A compatibilidade do projeto de pesquisa com as prioridades estratégicas da agência de fomento é um fator crítico para a aprovação do projeto de pesquisa.

◯ Concordo muito
◯ Concordo
◯ Não concordo nem discordo
◯ Discordo
◯ Discordo muito

h10. O projeto conter um plano de divulgação científica e popularização da ciência.

◯ Concordo muito
◯ Concordo
◯ Não concordo nem discordo
◯ Discordo
◯ Discordo muito

Bloco I – FUGA DE TALENTOS INSTITUCIONAIS

i1. Nos últimos cinco anos, você pensou ou tentou trabalhar em outra instituição no Brasil?

◯ Sim ◯ Não (Pular para a pergunta [i7])

Indique o quanto você concorda com as seguintes afirmativas sobre o motivo de você ter pensado ou tentado trabalhar em outra instituição no Brasil:

i2. Remuneração baixa.

◯ Concordo muito
◯ Concordo
◯ Não concordo nem discordo
◯ Discordo
◯ Discordo muito

i3. Dificuldades de obter financiamento para pesquisas.

◯ Concordo muito
◯ Concordo
◯ Não concordo nem discordo
◯ Discordo
◯ Discordo muito

i4. Falta de tempo/espaço para desenvolvimento de atividades de pesquisa.

◯ Concordo muito
◯ Concordo
◯ Não concordo nem discordo
◯ Discordo
◯ Discordo muito

i5. Para trabalhar com pesquisadores ou grupos de pesquisas consolidados.

◯ Concordo muito
◯ Concordo
◯ Não concordo nem discordo
◯ Discordo
◯ Discordo muito

i6. Sentimento de desvalorização como pesquisador.

◯ Concordo muito
◯ Concordo
◯ Não concordo nem discordo
◯ Discordo
◯ Discordo muito

i7. Nos últimos 5 anos, você pensou ou tentou trabalhar em uma instituição no exterior?

◯ Sim ◯ Não

Indique o quanto você concorda com as seguintes afirmativas sobre o motivo de você ter pensado ou tentado trabalhar em uma instituição no exterior:

i8. Remuneração baixa.

◯ Concordo muito
◯ Concordo
◯ Não concordo nem discordo
◯ Discordo
◯ Discordo muito

i9. Dificuldades de obter financiamento para pesquisas.

◯ Concordo muito
◯ Concordo
◯ Não concordo nem discordo
◯ Discordo
◯ Discordo muito

i10. Falta de oportunidades para trabalhar.

◯ Concordo muito
◯ Concordo
◯ Não concordo nem discordo
◯ Discordo
◯ Discordo muito

i11. Para trabalhar com pesquisadores ou grupos de pesquisas consolidados.

◯ Concordo muito
◯ Concordo
◯ Não concordo nem discordo
◯ Discordo
◯ Discordo muito

i12. Sentimento de desvalorização como pesquisador.

◯ Concordo muito
◯ Concordo
◯ Não concordo nem discordo
◯ Discordo
◯ Discordo muito

Bloco J – BOLSISTA DE PRODUTIVIDADE DO CNPQ

j1. Quantas vezes você já aplicou para uma bolsa de produtividade?

◯ Nenhuma ◯ Uma vez ◯ Duas vezes ◯ Três vezes ◯ Mais de três vezes

j2. Você é bolsista de produtividade do CNPq?

◯ Sim ◯ Não (Pular para a pergunta [j5])

j3. Qual o nível da sua bolsa de produtividade?

◯ Sênior ◯ 1A ◯ 1B ◯ 1C ◯ 1D ◯ 2

j4. Após quantos anos de conclusão do seu doutorado você foi contemplado com a bolsa de produtividade?

◯ 1 ano ◯ 2 anos ◯ 3 anos ◯ 4 anos ◯ 5 anos
◯ 6 anos ◯ 7 anos ◯ 8 anos ◯ 9 anos ◯ 10 anos
◯ Mais de 10 anos depois

j5. Se você está mesmo lendo esta pergunta, marque a opção “Azul”.

◯ Amarelo ◯ Azul ◯ Vermelho ◯ Branco ◯ Verde

Bloco K – USO DE REDES SOCIAIS

Segue abaixo uma lista de redes sociais frequentemente usadas no celular, computador ou tablet. Por favor, indique qual ou quais dessas você usa ou se você não usa nenhuma dessas redes sociais.

k1. WhatsApp
- ◯ Até 6 vezes por semana
- ◯ 1 a 3 vezes por semana
- ◯ 1 vez a cada 15 dias
- ◯ 1 vez por mês ou menos
- ◯ Não uso esta rede social

k2. Facebook
- ◯ Até 6 vezes por semana
- ◯ 1 a 3 vezes por semana
- ◯ 1 vez a cada 15 dias
- ◯ 1 vez por mês ou menos
- ◯ Não uso esta rede social

k3. Twitter
- ◯ Até 6 vezes por semana
- ◯ 1 a 3 vezes por semana
- ◯ 1 vez a cada 15 dias
- ◯ 1 vez por mês ou menos
- ◯ Não uso esta rede social

k4. Instagram
- ◯ Até 6 vezes por semana
- ◯ 1 a 3 vezes por semana
- ◯ 1 vez a cada 15 dias
- ◯ 1 vez por mês ou menos
- ◯ Não uso esta rede social

k5. LinkedIn
- ◯ Até 6 vezes por semana
- ◯ 1 a 3 vezes por semana
- ◯ 1 vez a cada 15 dias
- ◯ 1 vez por mês ou menos
- ◯ Não uso esta rede social

k6. YouTube
- ◯ Até 6 vezes por semana
- ◯ 1 a 3 vezes por semana
- ◯ 1 vez a cada 15 dias
- ◯ 1 vez por mês ou menos
- ◯ Não uso esta rede social

k7. ResearchGate

- ◯ Até 6 vezes por semana
- ◯ 1 a 3 vezes por semana
- ◯ 1 vez a cada 15 dias
- ◯ 1 vez por mês ou menos
- ◯ Não uso esta rede social

k8. Publons

- ◯ Até 6 vezes por semana
- ◯ 1 a 3 vezes por semana
- ◯ 1 vez a cada 15 dias
- ◯ 1 vez por mês ou menos
- ◯ Não uso esta rede social

k9. Você costuma divulgar suas pesquisas (artigos publicados, working papers, trabalhos apresentados em congressos etc.) em suas redes sociais?

◯ Sim (Pular para a pergunta [k11]) ◯ Não

k10. Por que não divulga suas pesquisas?

- ◯ Não acho que vale a pena
- ◯ Não tenho tempo
- ◯ Prefiro usar redes sociais para lazer
- ◯ Divulgação de pesquisas em redes sociais
- ◯ Outro: ____

k11. A instituição na qual você trabalha possui canal no YouTube?

◯ Sim ◯ Não sei ◯ Não

k12. Esse canal faz divulgação de pesquisas científicas (artigos publicados, working papers, trabalhos apresentados em congressos etc.)?

◯ Sim ◯ Não sei ◯ Não

k13. A instituição na qual você trabalha incentiva a divulgação dos trabalhos destacados na página ou publicação específica do Instituto para a comunidade?

◯ Sim ◯ Não sei ◯ Não

Bloco L – COOPERAÇÃO INTERNACIONAL

Segue abaixo uma lista de idiomas. Por favor, indique o seu grau de fluência para cada um desses idiomas.

l1. Português

◯ Muito fluente
◯ Fluente
◯ Razoavelmente fluente
◯ Pouco fluente
◯ Sem conhecimento do idioma

l2. Inglês

◯ Muito fluente
◯ Fluente
◯ Razoavelmente fluente
◯ Pouco fluente
◯ Sem conhecimento do idioma

l3. Espanhol

◯ Muito fluente
◯ Fluente
◯ Razoavelmente fluente
◯ Pouco fluente
◯ Sem conhecimento do idioma

l4. Francês

◯ Muito fluente
◯ Fluente
◯ Razoavelmente fluente
◯ Pouco fluente
◯ Sem conhecimento do idioma

l5. Alemão

◯ Muito fluente
◯ Fluente
◯ Razoavelmente fluente
◯ Pouco fluente
◯ Sem conhecimento do idioma

l6. Com que frequência você participa de conversas com pesquisadores ou professores colaboradores em línguas estrangeiras?

◯ Todos os dias
◯ Algumas vezes por semana
◯ Algumas vezes por mês
◯ Algumas vezes por ano
◯ Nunca

l7. Você já participou de cooperações internacionais (cooperação de sua instituição com instituições de outros países)?

◯ Sim ◯ Não (Pular para a pergunta [l12])

l8. De quantas cooperações internacionais você já participou?

◯ 1 ◯ 2 ◯ 3 ◯ 4 ◯ 5 ◯ 6 ou mais

l9. Escreva abaixo os países das instituições com as quais você já participou de cooperações internacional. (Ex.: França; Alemanha; e Finlândia)

____

____

____

l10. Foram publicados artigos a partir dessas cooperações?

◯ Sim ◯ Não

l11. Qual a forma de financiamento dessas cooperações? (Assinale todas as que se aplicarem).

◯ Bolsa de agência nacional
◯ Bolsa de agência internacional
◯ Editais nacionais de financiamento
◯ Fontes internacionais de financiamento
◯ Recursos próprios

l12. Nos últimos 5 anos você submeteu algum projeto para agências de fomento no exterior?

◯ Sim (Pular para a pergunta [m1]) ◯ Não

l13. Por que não submeteu projetos para agências de fomento no exterior nesses últimos cinco anos? (Marque todas que se aplicam).

◯ Falta de oportunidades
◯ Falta de apoio para conseguir a documentação necessária
◯ Dificuldade de identificação de chamadas
◯ Falta de conhecimento sobre as agências disponíveis
◯ Dificuldades linguísticas relacionadas ao idioma do país da agência escolhida
◯ Falta de colaboradores ou parceiros no exterior
◯ Outro: __________________________________

Neste momento estamos finalizando as questões referentes aos tópicos institucionais. As próximas perguntas serão sobre questões pessoais que possam impactar a rotina do pesquisador.

De agora em diante, mais 7 minutos e finalizaremos o nosso questionário.

Bloco M – ESTRUTURA FAMILIAR E O IMPACTO DA MATERNIDADE

m1. Você tem filhos?
◯ Sim ◯ Não (Pular para a questão [n1])

m2. Quantos filhos você tem?
◯ 1 ◯ 2 ◯ 3 ◯ 4 ◯ Mais de 4 filhos

m3. Qual a idade do seu filho mais novo?
◯ Menos de 1 ano
◯ 1 ano ◯ 7 anos ◯ 13 anos
◯ 2 anos ◯ 8 anos ◯ 14 anos
◯ 3 anos ◯ 9 anos ◯ 15 anos
◯ 4 anos ◯ 10 anos ◯ 16 anos
◯ 5 anos ◯ 11 anos ◯ 17 anos
◯ 6 anos ◯ 12 anos ◯ 18 anos ou mais

Pontue o impacto de ter filhos ou filhas em sua carreira acadêmica, em uma escala de 1 ("nenhum impacto") e 5 ("muito impacto"), nas seguintes atividades.

m4. Produção científica
◯ 1 ◯ 2 ◯ 3 ◯ 4 ◯ 5

m5. Atividades didáticas
◯ 1 ◯ 2 ◯ 3 ◯ 4 ◯ 5

m6. Credenciamento e recredenciamento em programas de pós-graudação
◯ 1 ◯ 2 ◯ 3 ◯ 4 ◯ 5

m7. Atividades de extensão
◯ 1 ◯ 2 ◯ 3 ◯ 4 ◯ 5

m8. Atividades de divulgação científica
◯ 1 ◯ 2 ◯ 3 ◯ 4 ◯ 5

m9. Participação em eventos internacionais
◯ 1 ◯ 2 ◯ 3 ◯ 4 ◯ 5

m10. Participação em eventos nacionais
◯ 1 ◯ 2 ◯ 3 ◯ 4 ◯ 5

m11. Atividades de orientação
◯ 1 ◯ 2 ◯ 3 ◯ 4 ◯ 5

m12. Formação de redes de colaboração
◯ 1 ◯ 2 ◯ 3 ◯ 4 ◯ 5

m13. Coordenação de projetos de pesquisa
◯ 1 ◯ 2 ◯ 3 ◯ 4 ◯ 5

m14. Liderança de grupo de pesquisa
◯ 1 ◯ 2 ◯ 3 ◯ 4 ◯ 5

Bloco N – QUALIDADE DE VIDA DO PESQUISADOR

Este questionário é sobre como você se sente a respeito de sua qualidade de vida, saúde e outras áreas de sua vida. Por favor, responda todas as questões. Se você não tem certeza sobre que resposta dar em uma questão, por favor, escolha entre as alternativas a que lhe parece mais apropriada. Esta, muitas vezes, poderá ser a sua primeira escolha.

Por favor, tenha em mente seus valores, aspirações, prazeres e preocupações. Nós estamos perguntando o que você acha de sua vida, tomando como referência às 2 ÚLTIMAS SEMANAS.

n1. Como você avaliaria sua qualidade de vida? (Por favor, leia cada questão, veja o que você acha e circule no número e lhe parece a melhor resposta).

◯ Muito ruim ◯ Ruim ◯ Nem ruim nem boa ◯ Boa ◯ Muito boa

n2. Quão satisfeito você está com a sua saúde?

◯ Muito insatisfeito
◯ Insatisfeito
◯ Nem satisfeito nem insatisfeito
◯ Satisfeito
◯ Muito satisfeito

As questões seguintes são sobre o quanto você tem sentido algumas coisas nas últimas 2 semanas.

n3. Em que medida você acha que sua dor (física) impede você de fazer o que você precisa?

◯ Nada ◯ Pouco ◯ Mais ou menos ◯ Bastante ◯ Extremamente

n4. O quanto você precisa de algum tratamento médico para levar sua vida diária?

◯ Nada ◯ Pouco ◯ Mais ou menos ◯ Bastante ◯ Extremamente

n5. O quanto você aproveita a vida?

◯ Nada ◯ Pouco ◯ Mais ou menos ◯ Bastante ◯ Extremamente

n6. Em que medida você acha que a sua vida tem sentido?

◯ Nada ◯ Pouco ◯ Mais ou menos ◯ Bastante ◯ Extremamente

n7. O quanto você consegue se concentrar?

◯ Nada ◯ Pouco ◯ Mais ou menos ◯ Bastante ◯ Extremamente

n8. O quanto você se sente em segurança em sua vida diária?

◯ Nada ◯ Pouco ◯ Mais ou menos ◯ Bastante ◯ Extremamente

n9. Quão saudável é o seu ambiente físico (clima, barulho, poluição, atrativos)?

◯ Nada ◯ Pouco ◯ Mais ou menos ◯ Bastante ◯ Extremamente

As questões seguintes perguntam sobre quão completamente você tem sentido ou é capaz de fazer certas coisas nestas últimas 2 semanas.

n10. Você tem energia suficiente para seu dia a dia?

◯ Nada ◯ Pouco ◯ Médio ◯ Muito ◯ Completamente

n11. Você é capaz de aceitar sua aparência física?

◯ Nada ◯ Pouco ◯ Médio ◯ Muito ◯ Completamente

n12. Você tem dinheiro suficiente para satisfazer suas necessidades?

◯ Nada ◯ Pouco ◯ Médio ◯ Muito ◯ Completamente

n13. Quão disponíveis para você estão as informações que precisa no seu dia a dia?

◯ Nada ◯ Pouco ◯ Médio ◯ Muito ◯ Completamente

n14. Em que medida você tem oportunidades de atividade de lazer?

◯ Nada ◯ Pouco ◯ Médio ◯ Muito ◯ Completamente

As questões seguintes perguntam sobre quão bem ou satisfeito você se sentiu a respeito de vários aspectos de sua vida nas últimas 2 semanas.

n15. Quão bem você é capaz de se locomover?

◯ Muito ruim ◯ Ruim ◯ Nem ruim nem bom ◯ Bom ◯ Muito bom

As questões seguintes perguntam sobre quão bem ou satisfeito você se sentiu a respeito de vários aspectos de sua vida nas últimas 2 semanas.

n16. Quão satisfeito você está com o seu sono?

◯ Muito insatisfeito
◯ Insatisfeito
◯ Nem satisfeito nem insatisfeito
◯ Satisfeito
◯ Muito satisfeito

n17. Quão satisfeito você está com sua capacidade de desempenhar as atividades do seu dia a dia?

◯ Muito insatisfeito
◯ Insatisfeito
◯ Nem satisfeito nem insatisfeito
◯ Satisfeito
◯ Muito satisfeito

n18. Quão satisfeito você está com sua capacidade para o trabalho?

◯ Muito insatisfeito
◯ Insatisfeito
◯ Nem satisfeito nem insatisfeito
◯ Satisfeito
◯ Muito satisfeito

n19. Quão satisfeito você está consigo mesmo?
◯ Muito insatisfeito
◯ Insatisfeito
◯ Nem satisfeito nem insatisfeito
◯ Satisfeito
◯ Muito satisfeito

n20. Quão satisfeito você está com suas relações pessoais (amigos, parentes, conhecidos, colegas)?
◯ Muito insatisfeito
◯ Insatisfeito
◯ Nem satisfeito nem insatisfeito
◯ Satisfeito
◯ Muito satisfeito

n21. Quão satisfeito você está com sua vida sexual?
◯ Muito insatisfeito
◯ Insatisfeito
◯ Nem satisfeito nem insatisfeito
◯ Satisfeito
◯ Muito satisfeito

n22. Quão satisfeito você está com o apoio que você recebe de seus amigos?
◯ Muito insatisfeito
◯ Insatisfeito
◯ Nem satisfeito nem insatisfeito
◯ Satisfeito
◯ Muito satisfeito

n23. Quão satisfeito você está com as condições do local onde mora?
◯ Muito insatisfeito
◯ Insatisfeito
◯ Nem satisfeito nem insatisfeito
◯ Satisfeito
◯ Muito satisfeito

n24. Quão satisfeito você está com o seu acesso aos serviços de saúde?
◯ Muito insatisfeito
◯ Insatisfeito
◯ Nem satisfeito nem insatisfeito
◯ Satisfeito
◯ Muito satisfeito

n25. Quão satisfeito você está com o seu meio de transporte?
◯ Muito insatisfeito
◯ Insatisfeito
◯ Nem satisfeito nem insatisfeito
◯ Satisfeito
◯ Muito satisfeito

As questões seguintes referem-se a com que frequência você sentiu ou experimentou certas coisas nas últimas 2 semanas.

n26. Com que frequência você tem sentimentos negativos como mau humor, desespero, ansiedade e depressão?

◯ Nunca ◯ Algumas vezes ◯ Frequentemente ◯ Muito frequente ◯ Sempre

n27. Este questionário fez você refletir sobre sua atuação enquanto pesquisador ou servidor do IF Goiano?

◯ Totalmente ◯ Muito ◯ Parcialmente ◯ Pouco ◯ Não fez refletir

n28. Há algum comentário final ou tópico não abordado neste questionário que você gostaria de mencionar?

Agradecemos muito sua participação!

Em breve compartilharemos os resultados da nossa pesquisa.
