# Supplementary material for: Research funding challenges in Brazil: researchers' perceptions from a public institution of professional education
Source: Front Res Metr Anal. 2025 Sep 22;10:1553928. doi: 10.3389/frma.2025.1553928 (PMC12497820; doi:10.3389/frma.2025.1553928)
Supplement: Supplementary file 9 [file Data_Sheet_5.pdf]

Supplementary Material S2

Survey questionnaire – English translation version

IF Goiano Researcher Profile

Dear Researcher,

We are researchers at the Instituto Federal Goiano (IF Goiano), and we are carrying out a survey on FUNDING IN SCIENTIFIC RESEARCH AND QUALITY OF LIFE OF THE RESEARCHER. The results may provide useful insights to guide best practices for researchers, funding agencies, and research institutions in the future. We count on your valuable participation.

Block A – GUEST DECISION

The estimated time to complete the questionnaire is approximately 15 minutes. Thank you very much in advance for your cooperation!

Email: ____

Informed Consent Form

This study was approved by the Research Ethics Committee (Protocol CAAE No. 67695523.4.0000.0036 and Opinion No. 6.144.987). Before you begin, please access the ICF to decide on your voluntary participation.

The ICF complies with item IV.5, paragraph "a" of CNS Resolution No. 466/2012, fulfilling the requirements of item IV.3. If you choose to participate, by selecting "AGREE" and submitting your answers, you give your informed and voluntary consent. Participation is essential for this study, but you are free to withdraw at any time, without penalty. By selecting "DISAGREE", there will be no penalty for you.

Having read and understood the Informed Consent Form (ICF), do you accept and agree to take part in this study?

◯ **Agree** ◯ **Disagree**

Providing the link to your CV Lattes will allow us to extract all of your scientific production, reducing the number of questions in this questionnaire and, consequently, optimizing your response time.

Please provide a link to your Lattes CV (optional): ____________________________________

Block B – SOCIODEMOGRAPHICAL DATA

b1. How old are you? __________ (in years)

b2. You identify with the genre:
◯ Female
◯ Male
◯ Non-binary
◯ I prefer not to answer
◯ Other: ______________________________________

b3. What is your skin color/ethnicity?
◯ Black ◯ Mixed ◯ White ◯ Asian ◯ Indigenous

b4. What is your marital status?
◯ Single
◯ Married or in a stable union
◯ Divorced or separated
◯ Widower

Block C – WORK-RELATED DATA

c1. Where do you work? (Indicate the main IF Goiano unit where you work or collaborate. If you work in more than one unit, select only the one you consider to be your main unit).
◯ Rectory - Goiânia
◯ Advanced Campus of Catalão
◯ Advanced Campus of Hidrolândia
◯ Advanced Campus of Ipamerí
◯ Campus Campos Belos
◯ Campus Ceres
◯ Campus Cristalina
◯ Campus Iporá
◯ Campus Trindade
◯ Campus Morrinhos
◯ Campus Rio Verde
◯ Campus Urutaí
◯ Innovation Hub

c2. Professional Identification
◯ Professor ◯ Administrative Staff

c3. What are your working hours at IF Goiano?
◯ Up to 20 hours per week
◯ More than 20hours up 40 hours per week
◯ More than 40 hours a week

c4. What is your relationship with IF Goiano?
◯ Permanent civil servant
◯ Temporary employee (temporary contract)
◯ External collaborator
◯ Other: ______________________________________

c5. How long have you worked at IF Goiano? __________ (in years)

c6. What is your gross monthly salary?

- ◯ Up to R$ 2,999.99
- ◯ R$ 3,000.00 to R$ 5,999.99
- ◯ R$ 6,000.00 to R$ 8,999.99
- ◯ R$ 9,000.00 to R$ 11,999.99
- ◯ R$ 12,000.00 to R$ 14,999.99
- ◯ R$ 15,000.00 to R$ 17,999.99
- ◯ R$ 18,000.00 or more
- ◯ I prefer not to answer

c7. Thinking about your monthly salary, would you say that you are:

- ◯ Very satisfied
- ◯ Satisfied
- ◯ Neither satisfied nor dissatisfied
- ◯ Dissatisfied
- ◯ Very dissatisfied

c8. What is your highest level of education to date?

- ◯ Undergraduate degree
- ◯ Postgraduate lato sensu (specialization level)
- ◯ Master's degree
- ◯ Doctorate
- ◯ Postdoctoral training

c9. Did you complete any degree outside Brazil (full or part-time)?

- ◯ No
- ◯ Yes, undergraduate degree
- ◯ Yes, postgraduate lato sensu (specialization level)
- ◯ Yes, master's degree
- ◯ Yes, doctorate
- ◯ Yes, postdoctoral training
- ◯ Other: ____

c10. In addition to your work as a professor or administrative staff, do you hold any other position or function at IF Goiano:

◯ Yes ◯ No (Skip to question [c12])

c11. Specify the other functions you perform at IF Goiano:

- ◯ Rector
- ◯ Pro-Rectory or General Directorate
- ◯ Directorate
- ◯ General Coordination, Management, or Deputy Directorate
- ◯ Coordination
- ◯ Unit Head
- ◯ Section Head
- ◯ Division Head
- ◯ Department Head
- ◯ Other: ____

c12. Apart from IF Goiano, do you work in other higher education institutions or in another education network?

◯ Yes ◯ No (Skip to question [c14])

c13. How many hours a week do you work in other higher education institutions or in another education network besides IF Goiano?

◯ Up to 20 hours per week
◯ More than 20 hours up 40 hours per week
◯ More than 40 hours a week

c14. In the last 5 years, how much time on average have you spent per week on teaching activities?

◯ Less than 5 h ◯ 5–10 h ◯ 11–20 h ◯ 21–30 h ◯ More than 30 h

c15. In the last 5 years, on average, how much time have you spent per week on research activities?

◯ Less than 5 h ◯ 5–10 h ◯ 11–20 h ◯ 21–30 h ◯ More than 30 h

c16. In the last 5 years, on average, how much time have you spent per week on outreach activities?

◯ Less than 5 h ◯ 5–10 h ◯ 11–20 h ◯ 21–30 h ◯ More than 30 h

Thinking about your work routine, please read the statements below and indicate whether you strongly agree, agree, neither agree nor disagree, disagree, or strongly disagree with each of them.

c17. I spend most of my time on administrative tasks.

◯ I strongly agree
◯ I agree
◯ I neither agree nor disagree
◯ I disagree
◯ I strongly disagree

c18. I have been able to progress quickly in my career.

◯ I strongly agree
◯ I agree
◯ I neither agree nor disagree
◯ I disagree
◯ I strongly disagree

c19. I have enough time to work on my articles.

◯ I strongly agree
◯ I agree
◯ I neither agree nor disagree
◯ I disagree
◯ I strongly disagree

c20. Pursuing a research career in Brazil is not worthwhile.

◯ I strongly agree
◯ I agree
◯ I neither agree nor disagree
◯ I disagree
◯ I strongly disagree

c21. I find it easy to obtain funding for my research.

◯ I strongly agree
◯ I agree
◯ I neither agree nor disagree
◯ I disagree
◯ I strongly disagree

c22. My teaching load is too high.

◯ I strongly agree
◯ I agree
◯ I neither agree nor disagree
◯ I disagree
◯ I strongly disagree

c23. My intellectual output is greatly hampered by other tasks.

◯ I strongly agree
◯ I agree
◯ I neither agree nor disagree
◯ I disagree
◯ I strongly disagree

c24. I feel very motivated to work.

◯ I strongly agree
◯ I agree
◯ I neither agree nor disagree
◯ I disagree
◯ I strongly disagree

c25. Do you teach in any stricto sensu postgraduate programs?

◯ Yes ◯ No

c26. How long have you been working as a researcher? __________ (in years)

c27. Do you take part in any research projects or groups?

◯ Yes ◯ No (Skip to question [c28])

c28. What role do you play in research groups? (Check the option that represents the most active role you play in the research groups in which you participate).

- ◯ Leader (responsible for coordinating and planning the group's research work)
- ◯ Researcher (graduate or postgraduate servant involved in the development of projects and the scientific, technological, and artistic production of the research group)
- ◯ Technician (servant responsible for providing technical support for research and innovation projects and for the group's scientific, technological, and artistic production)
- ◯ Student (student who actively participates in scientific and technological production linked to the lines of research under the guidance of researchers from the research group)
- ◯ External member (researcher, technician, or student with an active relationship at another educational institution who contributes to the development of projects and the scientific, technological, and artistic production of the research group)

c29. Do you feel encouraged to carry out research at IF Goiano?

- ◯ Yes
- ◯ Partially (sometimes)
- ◯ No
- ◯ I do not know, or I prefer not to answer

c30. At this institution, do you feel encouraged to publish your research results?

- ◯ Yes
- ◯ Partially (sometimes)
- ◯ No
- ◯ I do not know, or I prefer not to answer

c31. Are you in the habit of doing research to improve your teaching or administrative work?

- ◯ Yes, often
- ◯ Yes, sometimes
- ◯ Rarely
- ◯ No, never

c32. In your opinion, does IF Goiano offer adequate conditions for professors and administrative staff to develop skills and act in the production of knowledge?

- ◯ Yes
- ◯ Partially (sometimes)
- ◯ No
- ◯ I do not know, or I prefer not to answer

Block D – FACTORS ASSOCIATED WITH PROJECT SUBMISSION

d1. In the last 5 years, how many research projects have you submitted to funding calls?

- ◯ None (Skip to question [f1])
- ◯ From 1 to 3
- ◯ From 4 to 6
- ◯ From 7 to 9
- ◯ From 10 to 12
- ◯ From 13 to 15
- ◯ 16 or more

d2. What are the main reasons for your decision to submit research projects to funding calls? (Check all that apply).

- ◯ Possibility of obtaining funding to carry out the project
- ◯ Increasing the impact and visibility of research
- ◯ Access to additional resources and infrastructure
- ◯ Strengthening the academic curriculum
- ◯ Institutional encouragement to submit projects
- ◯ Other: ____

d3. What are the main difficulties faced when submitting research projects for funding? (Check all that apply).

- ◯ Complex requirements and criteria in calls for proposals
- ◯ Limited availability of funding resources
- ◯ Competition with other researchers
- ◯ Difficulty in finding institutional partnerships
- ◯ Difficulty in finding collaborating researchers
- ◯ Time and effort required to prepare the project
- ◯ Other: ____

d4. When you fail to submit to a call for proposals, even if you are interested, what is the main reason for not submitting?

- ◯ Lack of knowledge about available funding notices
- ◯ Difficulty in finding partnerships or collaborators for the project
- ◯ Time constraints for preparing and submitting projects
- ◯ Difficulty in meeting the criteria and requirements of calls for proposals
- ◯ Lack of guidance and institutional support for submitting projects
- ◯ Other: ____

Block E – FACTORS ASSOCIATED WITH PROJECT APPROVAL

e1. In the last 5 years, how many research projects have you submitted that have been approved for funding?

◯ None (Skip to question [g1])
◯ From 1 to 3 ◯ From 7 to 9
◯ From 4 to 6 ◯ 10 or more

e2. After how many submissions to calls for funding did you manage to finance your first research project?

◯ The first time ◯ From 4 to 5 ◯ From 8 to 9
◯ From 2 to 3 ◯ From 6 to 7 ◯ 10 or more

e3. What strategies do you use to increase the chances of your research projects being approved for funding? (Check all that apply).

◯ Seek institutional or collaborative partnerships
◯ Draw up a clear and well-structured project
◯ Adapt the project to the criteria and objectives of the calls for proposals
◯ Make revisions and adjustments based on previous feedback
◯ Demonstrate the relevance and potential impact of the project
◯ Other: ______________________________

e4. In your opinion, what are the main criteria that influence the approval of projects in funding calls? (Check all that apply).

◯ Scientific merit of the project
◯ Relevance and impact of the project on society
◯ Experience and qualifications of the researchers involved
◯ Technical and methodological feasibility of the project
◯ Alignment with the objectives and priorities of the call for proposals
◯ Outro: ______________________________

e5. In your opinion, what are the main challenges faced in obtaining projects approved for funding? (Check all that apply).

◯ Fierce competition with other projects
◯ Budget restrictions and limited availability of resources
◯ Complexity of requirements and criteria of the call for proposals
◯ Difficulty in finding collaborative partnerships
◯ Time and effort required to prepare the proposal
◯ Other: ______________________________

e6. What kind of institutional support do you think is needed to increase the chances of projects being approved for funding? (Check all that apply).

◯ Guidance on preparing proposals
◯ Financial resources for the counterpart or project costs
◯ Specific training on project preparation and calls for proposals
◯ Encouraging the formation of partnerships and collaborative networks
◯ Other: ______________________________

Block F – FACTORS ASSOCIATED WITH PROJECT NON-SUBMISSION

f1. In the last 5 years, which institutions have been responsible for funding your research projects? (List each institution separated by a comma).

____

____

____

f2. In the last 5 years, what was the total amount of funding you obtained for your research? (Write in the following format: R$ 70,000.00) ____________________

f3. What is the main reason why you have not yet submitted a research project to a call for proposals? (Then, skip to question [h1])

◯ Lack of knowledge about available funding notices
◯ Difficulty in finding partnerships or collaborators for the project
◯ Time constraints for preparing and submitting projects
◯ Difficulty in meeting the criteria and requirements of calls for proposals
◯ Lack of guidance and institutional support for submitting projects
◯ Other: ________________________________

Block G – FACTORS ASSOCIATED WITH PROJECT NON-APPROVAL

g1. What factors do you believe contributed to the non-approval of your research projects? (Select all that apply).

◯ The project proposal was not clearly formulated
◯ Lack of previous experience in the research area
◯ The project was considered to be of low relevance or impact by the funding agency
◯ Lack of adequate resources or infrastructure to carry out the project
◯ Difficulties in writing the proposal (e.g., scientific writing)
◯ The budget presented for the project was considered inadequate
◯ The project proposal did not include national partnerships
◯ The project proposal did not include international partnerships
◯ Other: ________________________________

Block H – FACTORS ASSOCIATED WITH SUBMISSION AND APPROVAL

Based on your experience with research project submissions and approvals by funding agencies, please indicate how much you agree with the following statements:

h1. The clarity of the research proposal is a critical factor in the approval of research projects.

◯ I strongly agree
◯ I agree
◯ I neither agree nor disagree
◯ I disagree
◯ I strongly disagree

h2. The relevance and originality of the research project are essential for its approval.

- ◯ I strongly agree
- ◯ I agree
- ◯ I neither agree nor disagree
- ◯ I disagree
- ◯ I strongly disagree

h3. The expertise of the researcher or research team is a significant factor in the approval of research projects.

- ◯ I strongly agree
- ◯ I agree
- ◯ I neither agree nor disagree
- ◯ I disagree
- ◯ I strongly disagree

The methodological design of the research project strongly influences its acceptance.

h4. The availability of resources (e.g., laboratory facilities, equipment, etc.) at the research site impacts the approval of the research project.

- ◯ I strongly agree
- ◯ I agree
- ◯ I neither agree nor disagree
- ◯ I disagree
- ◯ I strongly disagree

h5. The project's adherence to ethical guidelines is an important factor in the approval of a research project.

- ◯ I strongly agree
- ◯ I agree
- ◯ I neither agree nor disagree
- ◯ I disagree
- ◯ I strongly disagree

h6. The existence of partnerships or collaborations with other institutions increases the likelihood of a research project being approved.

- ◯ I strongly agree
- ◯ I agree
- ◯ I neither agree nor disagree
- ◯ I disagree
- ◯ I strongly disagree

h7. The proportion of the project budget for carrying out the research is an important factor in the approval of the research project.

- ◯ I strongly agree
- ◯ I agree
- ◯ I neither agree nor disagree
- ◯ I disagree
- ◯ I strongly disagree

h8. The demonstration of the significant impact of the proposed research (social, economic, environmental impact, etc.) affects the approval of the research project.

◯ I strongly agree
◯ I agree
◯ I neither agree nor disagree
◯ I disagree
◯ I strongly disagree

h9. The compatibility of the research project with the strategic priorities of the funding agency is a critical factor in the approval of the research project.

◯ I strongly agree
◯ I agree
◯ I neither agree nor disagree
◯ I disagree
◯ I strongly disagree

h10. The project contains a plan to disseminate science and popularize science.

◯ I strongly agree
◯ I agree
◯ I neither agree nor disagree
◯ I disagree
◯ I strongly disagree

Block I – INSTITUTIONAL TALENT DRIFT

i1. In the last five years, have you considered or tried working at another institution in Brazil?

◯ Yes ◯ No (Skip to question [i7])

Please indicate how much you agree with the following statements about why you have considered or tried to work at another institution in Brazil:

i2. Low pay.

◯ I strongly agree
◯ I agree
◯ I neither agree nor disagree
◯ I disagree
◯ I strongly disagree

i3. Difficulties in obtaining research funding.

◯ I strongly agree
◯ I agree
◯ I neither agree nor disagree
◯ I disagree
◯ I strongly disagree

i4. Lack of time/space for research activities.

- ◯ I strongly agree
- ◯ I agree
- ◯ I neither agree nor disagree
- ◯ I disagree
- ◯ I strongly disagree

i5. To work with established researchers or research groups.

- ◯ I strongly agree
- ◯ I agree
- ◯ I neither agree nor disagree
- ◯ I disagree
- ◯ I strongly disagree

i6. Feeling devalued as a researcher.

- ◯ I strongly agree
- ◯ I agree
- ◯ I neither agree nor disagree
- ◯ I disagree
- ◯ I strongly disagree

i7. In the last 5 years, have you thought about or tried to work in an institution abroad?

◯ Yes ◯ No

Please indicate how much you agree with the following statements about why you have thought about or tried to work in an institution abroad:

i8. Low pay.

- ◯ I strongly agree
- ◯ I agree
- ◯ I neither agree nor disagree
- ◯ I disagree
- ◯ I strongly disagree

i9. Difficulties in obtaining research funding.

- ◯ I strongly agree
- ◯ I agree
- ◯ I neither agree nor disagree
- ◯ I disagree
- ◯ I strongly disagree

i10. Lack of opportunities to work.

- ◯ I strongly agree
- ◯ I agree
- ◯ I neither agree nor disagree
- ◯ I disagree
- ◯ I strongly disagree

i11. To work with established researchers or research groups.

- ◯ I strongly agree
- ◯ I agree
- ◯ I neither agree nor disagree
- ◯ I disagree
- ◯ I strongly disagree

i12. Feeling devalued as a researcher.

- ◯ I strongly agree
- ◯ I agree
- ◯ I neither agree nor disagree
- ◯ I disagree
- ◯ I strongly disagree

Block J – CNPQ PRODUCTIVITY FELLOWSHIP

j1. How many times have you applied for a productivity fellowship?

◯ Never ◯ Once ◯ Twice ◯ Three times ◯ More than three times

j2. Are you a CNPq productivity fellowship holder?

◯ Yes ◯ No (Skip to question [j5])

j3. What level is your productivity fellowship?

◯ Senior ◯ 1A ◯ 1B ◯ 1C ◯ 1D ◯ 2

j4. How many years after completing your doctorate were you awarded the productivity fellowship?

◯ 1 year ◯ 2 years ◯ 3 years ◯ 4 years ◯ 5 years
◯ 6 years ◯ 7 years ◯ 8 years ◯ 9 years ◯ 10 years
◯ More than 10 years later

j5. If you are actually reading this question, check the "Blue" box.

◯ Yellow ◯ Blue ◯ Red ◯ White ◯ Green

Block K – USE OF SOCIAL NETWORKS

Below is a list of social networks that you frequently use on your cell phone, computer, or tablet. Please indicate which one or ones you use, or if you don't use any of these social networks.

k1. WhatsApp
- ◯ Up to 6 times a week
- ◯ 1 to 3 times a week
- ◯ 1 time every 15 days
- ◯ 1 time per month or less
- ◯ I do not use this social network

k2. Facebook
- ◯ Up to 6 times a week
- ◯ 1 to 3 times a week
- ◯ 1 time every 15 days
- ◯ 1 time per month or less
- ◯ I do not use this social network

k3. Twitter
- ◯ Up to 6 times a week
- ◯ 1 to 3 times a week
- ◯ 1 time every 15 days
- ◯ 1 time per month or less
- ◯ I do not use this social network

k4. Instagram
- ◯ Up to 6 times a week
- ◯ 1 to 3 times a week
- ◯ 1 time every 15 days
- ◯ 1 time per month or less
- ◯ I do not use this social network

k5. LinkedIn
- ◯ Up to 6 times a week
- ◯ 1 to 3 times a week
- ◯ 1 time every 15 days
- ◯ 1 time per month or less
- ◯ I do not use this social network

k6. YouTube
- ◯ Up to 6 times a week
- ◯ 1 to 3 times a week
- ◯ 1 time every 15 days
- ◯ 1 time per month or less
- ◯ I do not use this social network

k7. ResearchGate

◯ Up to 6 times a week
◯ 1 to 3 times a week
◯ 1 time every 15 days
◯ 1 time per month or less
◯ I do not use this social network

k8. Publons

◯ Up to 6 times a week
◯ 1 to 3 times a week
◯ 1 time every 15 days
◯ 1 time per month or less
◯ I do not use this social network

k9. Do you usually share your research (published articles, working papers, papers presented at conferences, etc.) on your social networks?

◯ Yes (Skip to question [k11]) ◯ No

k10. Why do not you publish your research?

◯ I do not consider it worthwhile
◯ I do not have enough time
◯ I prefer to use social media for leisure purposes
◯ Research dissemination on social media
◯ Other: ______________________________

k11. Does the institution you work for have a YouTube channel?

◯ Yes ◯ I do not know ◯ No

k12. Does this channel disseminate scientific research (published articles, working papers, papers presented at congresses, etc.)?

◯ Yes ◯ I do not know ◯ No

k13. Does the institution where you work encourage the dissemination of the work highlighted on the Institute's website or a specific publication to the community?

◯ Yes ◯ I do not know ◯ No

Block L – INTERNATIONAL COOPERATION

Below is a list of languages. Please indicate your degree of fluency for each of these languages.

l1. Portuguese

◯ Very fluent
◯ Fluent
◯ Reasonably fluent
◯ Slightly fluent
◯ No knowledge of the language

l2. English

◯ Very fluent
◯ Fluent
◯ Reasonably fluent
◯ Slightly fluent
◯ No knowledge of the language

l3. Spanish

◯ Very fluent
◯ Fluent
◯ Reasonably fluent
◯ Slightly fluent
◯ No knowledge of the language

l4. French

◯ Very fluent
◯ Fluent
◯ Reasonably fluent
◯ Slightly fluent
◯ No knowledge of the language

l5. German

◯ Very fluent
◯ Fluent
◯ Reasonably fluent
◯ Slightly fluent
◯ No knowledge of the language

l6. How often do you participate in conversations with researchers or collaborating professors in foreign languages?

◯ Every day
◯ A few times a week
◯ A few times a month
◯ A few times a year
◯ Never

l7. Have you participated in international cooperation (cooperation between your institution and institutions in other countries)?

◯ Yes ◯ No (Skip to question [l12])

l8. How many international cooperation projects have you taken part in?

◯ 1 ◯ 2 ◯ 3 ◯ 4 ◯ 5 ◯ 6 or more

l9. Write down the countries of the institutions with which you have participated in international cooperation (e.g., France, Germany, and Finland).

____

____

____

____

____

____

____

l10. Have any articles been published from these cooperations?

◯ Yes ◯ No

l11. How are these cooperations financed? (Check all that apply).

◯ National agency grant
◯ International agency grant
◯ National funding calls for proposals
◯ International sources of funding
◯ Own resources

l12. Have you submitted any projects to funding agencies abroad in the last 5 years?

◯ Yes (Skip to question [m1]) ◯ No

l13. Why have you not submitted any projects to funding agencies abroad in the last five years? (Check all that apply).

◯ Lack of opportunities
◯ Lack of support to obtain the necessary documentation
◯ Difficulty identifying calls
◯ Lack of knowledge about available agencies
◯ Language difficulties related to the language of the country of the chosen agency
◯ Lack of collaborators or partners abroad
◯ Other: ______________________________

We are currently finalizing the questions on institutional topics.
The next questions will be about personal issues that may have an impact on the researcher's routine.

From now on, 7 more minutes and we will finish our questionnaire.

Block M – FAMILY STRUCTURE AND THE IMPACT OF PARENTHOOD

m1. Do you have children?

◯ Yes ◯ No (Skip to question [n1])

m2. How many children do you have?

◯ 1 ◯ 2 ◯ 3 ◯ 4 ◯ More than 4 children

m3. How old is your youngest child?

◯ Less than 1 year

◯ 1 year	◯ 7 years	◯ 13 years
◯ 2 years	◯ 8 years	◯ 14 years
◯ 3 years	◯ 9 years	◯ 15 years
◯ 4 years	◯ 10 years	◯ 16 years
◯ 5 years	◯ 11 years	◯ 17 years
◯ 6 years	◯ 12 years	◯ 18 years or older

Score the impact of having sons or daughters on your academic career, on a scale of 1 ("no impact") to 5 ("a lot of impact"), on the following activities.

m4. Scientific production

◯ 1 ◯ 2 ◯ 3 ◯ 4 ◯ 5

m5. Didactic activities

◯ 1 ◯ 2 ◯ 3 ◯ 4 ◯ 5

m6. Accreditation and re-accreditation in postgraduate programs

◯ 1 ◯ 2 ◯ 3 ◯ 4 ◯ 5

m7. Extension activities

◯ 1 ◯ 2 ◯ 3 ◯ 4 ◯ 5

m8. Scientific dissemination activities

◯ 1 ◯ 2 ◯ 3 ◯ 4 ◯ 5

m9. Participation in international events

◯ 1 ◯ 2 ◯ 3 ◯ 4 ◯ 5

m10. Participation in national events

◯ 1 ◯ 2 ◯ 3 ◯ 4 ◯ 5

m11. Orientation activities

◯ 1 ◯ 2 ◯ 3 ◯ 4 ◯ 5

m12. Collaborative networking

◯ 1 ◯ 2 ◯ 3 ◯ 4 ◯ 5

m13. Coordination of research projects

◯ 1 ◯ 2 ◯ 3 ◯ 4 ◯ 5

m14. Research group leadership

◯ 1 ◯ 2 ◯ 3 ◯ 4 ◯ 5

Block N – RESEARCHER QUALITY OF LIFE

This questionnaire is about how you feel about your quality of life, health, and other areas of your life. Please answer all the questions. If you are not sure which answer to give to a question, please choose the one that seems most appropriate. This can often be your first choice.

Please bear in mind your values, aspirations, pleasures, and concerns. We are asking you how you feel about your life, with reference to the LAST 2 WEEKS.

n1. How would you rate your quality of life? (Please read each question, see what you think, and circle the number that you think is the best answer).

◯ Very bad ◯ Bad ◯ Neither bad nor good ◯ Good ◯ Very good

n2. How satisfied are you with your health?

◯ Very dissatisfied
◯ Dissatisfied
◯ Neither satisfied nor dissatisfied
◯ Satisfied
◯ Very satisfied

The following questions are about how you have been feeling over the last 2 weeks.

n3. To what extent do you think your (physical) pain prevents you from doing what you need to do?

◯ Nothing ◯ Little ◯ More or less ◯ Quite a lot ◯ Extremely

n4. How much medical treatment do you need to go about your daily life?

◯ Nothing ◯ Little ◯ More or less ◯ Quite a lot ◯ Extremely

n5. How much do you enjoy life?

◯ Nothing ◯ Little ◯ More or less ◯ Quite a lot ◯ Extremely

n6. To what extent do you think your life has meaning?

◯ Nothing ◯ Little ◯ More or less ◯ Quite a lot ◯ Extremely

n7. How well can you concentrate?

◯ Nothing ◯ Little ◯ More or less ◯ Quite a lot ◯ Extremely

n8. How safe do you feel in your daily life?

◯ Nothing ◯ Little ◯ More or less ◯ Quite a lot ◯ Extremely

n9. How healthy is your physical environment (climate, noise, pollution, attractions)?

◯ Nothing ◯ Little ◯ More or less ◯ Quite a lot ◯ Extremely

The following questions ask how completely you have felt or been able to do certain things in the last 2 weeks.

n10. Do you have enough energy for your daily life?
◯ Nothing ◯ Little ◯ Medium ◯ A lot ◯ Completely

n11. Are you able to accept your physical appearance?
◯ Nothing ◯ Little ◯ Medium ◯ A lot ◯ Completely

n12. Do you have enough money to meet your needs?
◯ Nothing ◯ Little ◯ Medium ◯ A lot ◯ Completely

n13. How available is the information you need in your daily life?
◯ Nothing ◯ Little ◯ Medium ◯ A lot ◯ Completely

n14. To what extent do you have opportunities for leisure activities?
◯ Nothing ◯ Little ◯ Medium ◯ A lot ◯ Completely

The following questions ask how good or satisfied you have felt about various aspects of your life in the last 2 weeks.

n15. How well can you get around?
◯ Very bad ◯ Bad ◯ Neither bad nor good ◯ Good ◯ Very good

The following questions ask how good or satisfied you have felt about various aspects of your life in the last 2 weeks.

n16. How satisfied are you with your sleep?
◯ Very dissatisfied
◯ Dissatisfied
◯ Neither satisfied nor dissatisfied
◯ Satisfied
◯ Very satisfied

n17. How satisfied are you with your ability to carry out your day-to-day activities?
◯ Very dissatisfied
◯ Dissatisfied
◯ Neither satisfied nor dissatisfied
◯ Satisfied
◯ Very satisfied

n18. How satisfied are you with your ability to work?
◯ Very dissatisfied
◯ Dissatisfied
◯ Neither satisfied nor dissatisfied
◯ Satisfied
◯ Very satisfied

n19. How satisfied are you with yourself?

- ◯ Very dissatisfied
- ◯ Dissatisfied
- ◯ Neither satisfied nor dissatisfied
- ◯ Satisfied
- ◯ Very satisfied

n20. How satisfied are you with your personal relationships (friends, relatives, acquaintances, colleagues)?

- ◯ Very dissatisfied
- ◯ Dissatisfied
- ◯ Neither satisfied nor dissatisfied
- ◯ Satisfied
- ◯ Very satisfied

n21. How satisfied are you with your sex life?

- ◯ Very dissatisfied
- ◯ Dissatisfied
- ◯ Neither satisfied nor dissatisfied
- ◯ Satisfied
- ◯ Very satisfied

n22. How satisfied are you with the support you receive from your friends?

- ◯ Very dissatisfied
- ◯ Dissatisfied
- ◯ Neither satisfied nor dissatisfied
- ◯ Satisfied
- ◯ Very satisfied

n23. How satisfied are you with the conditions where you live?

- ◯ Very dissatisfied
- ◯ Dissatisfied
- ◯ Neither satisfied nor dissatisfied
- ◯ Satisfied
- ◯ Very satisfied

n24. How satisfied are you with your access to health services?

- ◯ Very dissatisfied
- ◯ Dissatisfied
- ◯ Neither satisfied nor dissatisfied
- ◯ Satisfied
- ◯ Very satisfied

n25. How satisfied are you with your means of transportation?

- ◯ Very dissatisfied
- ◯ Dissatisfied
- ◯ Neither satisfied nor dissatisfied
- ◯ Satisfied
- ◯ Very satisfied

The following questions refer to how often you have felt or experienced certain things in the last 2 weeks.

n26. How often do you experience negative feelings such as low mood, despair, anxiety, and depression?

◯ Never ◯ Sometimes ◯ Often ◯ Very frequent ◯ Always

n27. Has this questionnaire made you reflect on your work as a researcher or an IF Goiano employee?

◯ Totally ◯ A lot ◯ Partially ◯ Little ◯ It did not make you think

n28. Are there any final comments or topics not covered in this questionnaire that you would like to mention?

____

____

____

____

____

____

____

____

____

____

____

____

____

We truly appreciate your participation!

We will share the results of our survey shortly.
